# Immunological differences in atopic dermatitis across age groups: insights from single-cell multi-omics

**DOI:** 10.1186/s12967-026-08288-7

**Published:** 2026-06-01

**Authors:** Gian Carlo L. Baldonado, Sugandh Kumar, Joy Jin, Xiaohui Fang, Alexander Ildardashty, Mitchell Braun, Isaac M. Neuhaus, Erin Mathes, Tina Bhutani, Wilson Liao

**Affiliations:** 1 Department of Dermatology, University of California, 2340 Sutter Street, San Francisco, CA 94115 USA; 2 The Ronald O. Perelman Department of Dermatology, New York University Grossman School of Medicine, New York, NY USA; 3 Sackler School of Medicine, New York State/American Program, Tel Aviv University, Tel Aviv, Israel

**Keywords:** Atopic dermatitis, Single-cell multi-omics, Peripheral blood, Age differences, Immunology, CITE-seq, Transcriptomics, Proteomics, Machine learning

## Abstract

**Background:**

Atopic dermatitis (AD) occurs across all ages but presents distinct clinical and immunologic features between children, adults, and older adults. The molecular programs underlying these age-specific immune differences remain poorly understood.

**Methods:**

We performed single-cell multi-omics profiling of peripheral blood mononuclear cells (PBMCs) from 29 AD patients and 29 matched healthy controls (HC), spanning pediatric (0–17 years), adult (18–59 years), and geriatric (≥60 years) groups. Using Cellular Indexing of Transcriptomes and Epitopes by sequencing (CITE-seq), we simultaneously quantified transcriptomic (RNA) and surface proteomic (ADT) profiles across ~280,000 immune cells. Integrated analyses identified 30 immune subsets for cell-type proportion and differential expression analyses. Machine-learning classifiers were trained on significant gene and protein features to distinguish AD subgroups by age.

**Results:**

Compared with HC, AD blood showed enrichment of CD14+ monocytes, plasmacytoid dendritic cells, and CD4+ proliferating T cells, and differential gene expression analysis of AD vs HC revealed downstream Th2-associated signatures shared across all age groups. Within AD, pediatric patients had increased γδ T cells, naïve CD4+, and naïve CD8+ T cells, while geriatric patients exhibited more CD4+ cytotoxic and CD8+ central memory T cells, indicating a shift from naive to effector predominance with aging. Transcriptomic and proteomic analyses revealed distinct programs: pediatric AD was enriched for IL-10 and cytokine–cytokine receptor signaling; adult AD demonstrated activation of metabolic and nuclear factor kappa-light-chain-enhancer of activated B cells (NF-κB)/Th1/Th17 pathways; and geriatric AD exhibited reduced adaptive immune activity but increased innate signaling. Machine-learning models based on differentially expressed genes and proteins accurately classified AD age groups (transcript-based F1 = 0.70, AUC = 0.79), identifying stable markers such as IRF2, PDK4, ZFP90, CD21, CD94, and CD122.

**Conclusions:**

Single-cell multi-omics profiling revealed age-specific transcriptional and immunological programs overlaid on a shared Th2-driven inflammatory foundation in AD. Rather than discrete disease states, pediatric, adult, and geriatric AD each exhibited distinct molecular signatures: developmental and cytoskeletal in children, stress-response and chronic inflammatory in adults, and innate and metabolic in geriatric individuals. These findings support age-group molecular subtyping and age-tailored therapeutic strategies across the AD lifespan.

**Supplementary information:**

The online version contains supplementary material available at 10.1186/s12967-026-08288-7.

## Introduction

Atopic dermatitis (AD) is a chronic, inflammatory skin disease that affects up to 20% of children and 5–10% of adults worldwide [1, 2]. Most studies of AD pathogenesis have focused on skin-localized inflammation, revealing epidermal barrier dysfunction, immune polarization, and microbial imbalance as central drivers of disease [1, 3, 4]. Pediatric AD is typically characterized by T helper 2 (Th2) and T helper 22 (Th22) cytokine dominance, marked by elevated interleukin (IL)-4, IL-13, and IL-31 expression. With age, AD develops stronger T helper 1 (Th1) and T helper 17 (Th17) activity, reflecting broader immune activation and disease chronicity [3, 5]. Rather than replacing this Th2/Th22 inflammatory foundation, these age-specific immune programs are progressively superimposed upon a shared baseline that persists across the AD lifespan [3].

However, AD represents not only a skin barrier-centered disorder but also a systemic immune condition that involves coordinated dysregulation across circulating lymphoid and myeloid compartments [2]. Few studies have investigated peripheral blood mononuclear cells (PBMCs) at single-cell resolution, and age-stratified systemic analyses remain limited. Single-cell peripheral blood profiling of adult AD has revealed expanded interferon- and Janus kinase–signal transducer and activator of transcription (JAK–STAT)-activated monocytes, reduced natural killer (NK)-cell cytotoxicity, and increased co-stimulatory signaling, supporting the concept of systemic immune remodeling [4]. Comparative analyses of pediatric and adult AD have also identified Th2 predominance in children and Th2 dominance with increased Th1, Th17, and Th22 activity in adults, reflecting age-dependent immune divergence; however, these studies excluded older adults and were limited to transcriptomic data [3]. This gap highlights the need for additional studies examining age-specific assessments of systemic immunity in AD. Here, we performed single-cell multi-omics analysis integrating transcriptomic and proteomic profiling of peripheral blood from pediatric, adult, and geriatric patients and matched healthy controls to delineate age-dependent immune remodeling and identify predictive molecular signatures through integrative machine-learning analysis.

## Materials and Methods

### Subject recruitment and sampling

Subjects with atopic dermatitis (AD) were recruited from dermatology clinics at the University of California, San Francisco (UCSF). Healthy controls, matched by age, sex, and race/ethnicity, were recruited at UCSF. A total of 61 AD and healthy subjects were enrolled, stratified across three age groups: pediatric (0–17 years), adult (18–59 years), and geriatric (≥60 years). All AD diagnoses were confirmed by a board-certified dermatologist, and subjects were required to have at least moderate disease severity (validated Investigator Global Assessment for Atopic Dermatitis [vIGA-AD] ≥3). Exclusion criteria included current or recent use (within three months) of systemic immunomodulators, active autoimmune disease, active infection, or vaccination within the previous month. Clinical and demographic data collected included age, gender, race/ethnicity, body mass index, and vIGA-AD. Demographic characteristics are given in Supplementary Table 1, Additional File 1. Peripheral venous blood was collected for CITE-seq profiling of PBMCs.

### Sample and library preparation

#### Single-cell libraries

Peripheral blood was collected in Acid Citrate Dextrose (ACD) Vacutainer tubes and processed within 4 hours of phlebotomy. PBMCs were isolated by Ficoll gradient centrifugation, washed, and cryopreserved in 90% fetal bovine serum (FBS) + 10% dimethyl sulfoxide (DMSO) until use. For CITE-seq, 1 × 10^6^ cryopreserved PBMCs per subject were thawed, counted on a Vi-CELL XR (Beckman Coulter), and pooled in batches of ten subjects balanced by condition and age group. Following Fc blocking (Human TruStain FcX, BioLegend), cells were stained with a 140-plex TotalSeq-B antibody panel (Supplementary Table 2, Additional File 2) for 30 min at 4 °C, washed three times with Cell Staining Buffer, and filtered through 40 µm strainers. Cell viability exceeded 85% in all preparations.

#### Cell capture and library construction

4 × 10^5^ stained cells were loaded across 8 wells of a 10x Chromium Single Cell 3’ v3.1 chip, targeting approximately 2 × 10^5^ cell-containing droplets per run. Each droplet encapsulated a single cell for reverse transcription and barcode tagging.

Following lysis, cDNA libraries were prepared according to the manufacturer’s protocol, with separate size selection for gene-expression and antibody-derived tag (ADT) fragments. Libraries underwent amplification using KAPA HiFi HotStart ReadyMix (Kapa Biosystems) and size purification with SPRIselect reagent (Beckman Coulter). Final libraries were quantified by Bioanalyzer 2100 (Agilent) and sequenced on an Illumina NovaSeq 6000 (S4 flow cell) with a target depth of ~20,000– 40,000 reads/cell (RNA) and 5,000– 10,000 reads/cell (ADT).

### Genotyping

Genomic DNA was extracted from PBMCs using the DNeasy Blood and Tissue Kit (Qiagen). DNA samples were genotyped using the Affymetrix UK Biobank Axiom Array (ThermoFisher) on a GeneTitan Multi-Channel Instrument (Applied Biosystems). This genotype data was used to enable single-cell demultiplexing (see below).

#### Genotype data processing

Protocol for genotyping followed Liu et al [6]. Briefly, single nucleotide polymorphisms (SNPs) with call rate < 95%, Hardy-Weinberg *p* < 1 × 10^−5^, or minor allele frequency < 5% were removed. Imputation of non-genotyped sites was performed on the Michigan Imputation Server (1000 Genomes Phase 3 v5 Genome Reference Consortium Human build 37 (GRCh37) reference panel; Eagle v2.4 phasing). Next, variant call format (VCF) files were processed using Plink 1.90/2.0, with non-exonic SNPs removed and allele orientation verified using snpflip. Resulting high-quality genotype sets were converted to GRCh38 coordinates using Picard LiftoverVcf. Finally, imputed, quality-filtered genotype data were used as input for Demuxlet to assign each droplet barcode to its subject of origin.

### Single-cell data processing

Raw FASTQ files for RNA and ADT libraries were aligned to the GRCh38 human genome and the ADT feature barcode reference using Cell Ranger v8.0.1 of 10x Genomics with default parameters. Resulting transcript RNA and protein-expression count matrices were further processed by RStudio using Seurat v6. Only droplets identified as non-empty (cell-containing) were retained for downstream analysis.

#### Cell demultiplexing, doublet removal, and annotation

Demultiplexing was performed using Demuxlet (Popscle suite) [7], leveraging the imputed SNP genotypes to assign each droplet to its donor and remove inter-individual doublets. Remaining intra-sample doublets were removed using DoubletFinder (v2.0) based on simulated multiplet profiles. For each library, cell-level quality control (QC) metrics, including nCount_RNA, nFeature_RNA, and percent mitochondrial RNA, were evaluated before merging.

### Quality control and cell type annotation

Cells were retained if they met RNA and ADT quality thresholds. RNA-based filtering required nFeature_RNA ≥ 200, nCount_RNA within the interquartile range, and mitochondrial gene content ≤ 15%. For the ADT modality, cells were retained if total detected protein features were ≤ 260 and if isotype control signals contributed < 2% of total reads. RNA counts were normalized using SCTransform, regressing out technical covariates including sequencing depth (nCount_RNA) and mitochondrial content (percent.mt). ADT counts were normalized using the centered log-ratio (CLR) transformation.

Cell type annotation was performed using the Azimuth reference-mapping pipeline integrated into Seurat v6, which projects query datasets onto the Human Cell Atlas PBMC reference (Hao et al., 2021). SCTransform-normalized data were projected to the reference to assign prediction scores and cell identities through anchor-based mapping. Predicted labels were reviewed and refined through expert inspection of canonical RNA and ADT marker expression, ensuring biologically coherent annotation across modalities. This approach identified 30 major immune populations encompassing lymphoid and myeloid lineages.

### Outlier sample removal

Following QC and initial uniform manifold approximation and projection (UMAP), a cluster of CD14+ monocytes was observed adjacent to the primary CD14+ monocyte cluster (Supplementary Figures 1A-1C, Additional File 3). This island consisted almost exclusively of three samples (two pediatric AD and one adult AD) (Supplementary Figure 1D, Additional File 3), suggesting a sample-specific artifact rather than a true biological subset. To further characterize these samples, we examined standard QC metrics and cell type composition. Standard QC metrics, including nFeature_RNA, nCount_RNA, and mitochondrial gene percentage, did not reveal gross abnormalities in these samples; however, cells within the island showed notably higher medians for nFeature_RNA and nCount_RNA compared to cells from the same samples outside the island, and cell type composition confirmed a disproportionately high CD14+ monocyte proportion (Figures 2A–E, Additional File 4). These outlier samples were excluded prior to Harmony [8] integration, yielding a final dataset comprising 11 pediatric AD/HC, 13 adult AD/HC, and 5 geriatric AD/HC participants.

### Data integration

To correct for batch effects and align cells across sequencing runs, data integration was performed using Harmony implemented in Seurat v6. Principal component analysis (PCA) was conducted on SCTransform-normalized RNA expression, and the first 30 principal components were supplied to Harmony (RunHarmony, reduction = “pca”, dims = 1:30) with the *orig.ident* metadata variable as the grouping factor. The resulting Harmony embeddings were used to construct a shared nearest-neighbor (SNN) graph (FindNeighbors, reduction = “harmony”, dims = 1:30), identify clusters (FindClusters, resolution = 0.5), and generate UMAP visualizations (RunUMAP, reduction = “harmony”, dims = 1:30).

### Cell type proportion analysis

Cell type frequencies were calculated at the subject level as the proportion of each annotated cell type relative to the total number of singlet cells. For comparisons between AD and healthy controls, the Wilcoxon rank-sum test was applied. For three-group comparisons among pediatric, adult, and geriatric AD cohorts, both Kruskal–Wallis and pairwise Wilcoxon tests were performed. The Kruskal–Wallis test provided a global assessment of distributional differences across age groups, whereas pairwise Wilcoxon tests identified specific contrasts. Nominal *p*-values were reported, and cell-type proportions were visualized as grouped bar plots displaying mean ± standard error of the mean (SEM).

### Differential gene and protein expression

Differential expression analyses were conducted separately for RNA (genes) and ADT (proteins) within each annotated cell type to identify molecular features associated with 1) AD (all ages combined) vs healthy control (all ages combined) and 2) between different AD age groups. Three intra-AD contrasts were tested independently: pediatric AD vs (adult AD + geriatric AD), adult AD vs (pediatric AD + geriatric AD), and geriatric AD vs (pediatric AD + adult AD). For RNA, differential expression was assessed using the Seurat FindMarkers function on the SCTransform-normalized assay with the Wilcoxon rank-sum test and significance thresholds of |log₂FC| ≥0.5 and false discovery rate (FDR) <0.05. For proteins, differential expression used the CLR-normalized ADT assay with thresholds of log₂FC ≥ 0 and FDR < 0.05. No additional normalization was applied within subsets beyond global preprocessing. To visualize representative molecular programs across age groups, sentinel genes and proteins were selected from cell types with the highest numbers of significant DEGs and DEPs. For the most variable cell types, the top three features ranked by absolute log₂ fold-change were compiled. To ensure the resulting matrices captured biologically consistent and interpretable variation, features were retained only if they contained ≥ 2 non-missing (non-NA) log₂ fold-change values across (cell type × comparison) combinations and exhibited non-zero variance across comparisons.

### Machine learning model development

Subject-level feature matrices were generated from the mean normalized expression of significant, differentially expressed genes and proteins. For RNA-based models, genes meeting significance thresholds (|log₂FC| ≥0.50 and FDR < 0.05) were selected from the intra-AD comparisons, and their SCTransform-normalized z-scores were averaged per subject within each cell type. For protein-based models, significant proteins (FDR < 0.05) were selected from ADT differential analyses, and CLR-normalized z-scores were averaged per subject within each cell type. These per-subject means were concatenated across all cell types to produce DEG- and DEP-based subject matrices. Subjects were stratified by age group and divided into 60% training and 40% validation partitions. To prevent data leakage, all preprocessing—including median imputation and scaling—was performed exclusively on the training set, and the resulting parameters were applied to the held-out validation data.

Feature discovery was performed using a random-forest one-vs-rest importance framework, in which MeanDecreaseAccuracy scores were computed across 5 × 3 repeated cross-validation folds within the training partition. Normalized importance scores were aggregated across folds, and the top 20 features were frozen for model evaluation. Model development employed nested internal cross-validation for hyperparameter tuning within the training set, followed by evaluation on the single 40% validation split. Eight supervised algorithms were implemented using the *caret* framework: random forest (rf), linear support vector machine (svmLinear), elastic net (glmnet), k-nearest neighbors (knn), naïve Bayes, linear discriminant analysis (lda), shallow neural networks (nnet), and averaged neural networks (avNNet). Hyperparameters were tuned to optimize predictive performance while constraining model complexity. For random forest, parameters included *mtry*, *ntree = 800*, and *nodesize = 3*; for linear SVM, the cost parameter *C* ranged from 0.01 to 10; for elastic net, *α* (0–0.5) and *λ* (1e^−4^ −1) were varied; for k-NN, *k* values ranged from 5 to 11; for naïve Bayes, *laplace* (0–1) and *usekernel* (TRUE/FALSE) were tested; and for neural networks, hidden-layer size (1–2) and weight decay (0.01–0.1) were tuned with bagging disabled.

To address class imbalance and reduce variance, inverse-frequency class and case weights were applied according to model compatibility (e.g., *classwt* for random forest, *class.weights* for SVM, *weights* for neural networks). Overfitting was mitigated through model regularization, constrained tuning ranges, repeated cross-validation within the training partition, and the freezing of feature sets prior to validation. Model performance was evaluated using macro-averaged F1 as the primary metric, with area under the receiver operating characteristic curve (AUC) (one-vs-rest), overall accuracy, and Cohen’s κ as secondary measures. Random seeds (*set.seed = 742*) were fixed to ensure reproducibility. All analyses were conducted in R (v4.x) using *caret* and related packages.

## Results

### Clinical characteristics of subjects

After removing sample outliers, our study cohort included 58 participants across pediatric (0–17 years), adult (18–59 years), and geriatric (60+ years) age groups, with each group comprising both HC and individuals with AD (Supplementary Table 1, Additional File 1). The mean ages were 12.93 ± 4.84 (HC) and 12.36 ± 2.91 (AD) years for the pediatric group, 36.23 ± 13.23 and 36.00 ± 13.28 years for adults, and 70.60 ± 5.90 and 71.60 ± 5.94 years for the geriatric participants, respectively. Sex distribution was balanced across groups (27 females and 31 males overall). The cohort was racially and ethnically diverse, with representation from White, Asian, Black/African American, Hispanic/Latino, and Native Hawaiian/Pacific Islander participants.

### Cell types enriched and depleted in AD and among pediatric, adult, and geriatric AD

**Fig. 1:**
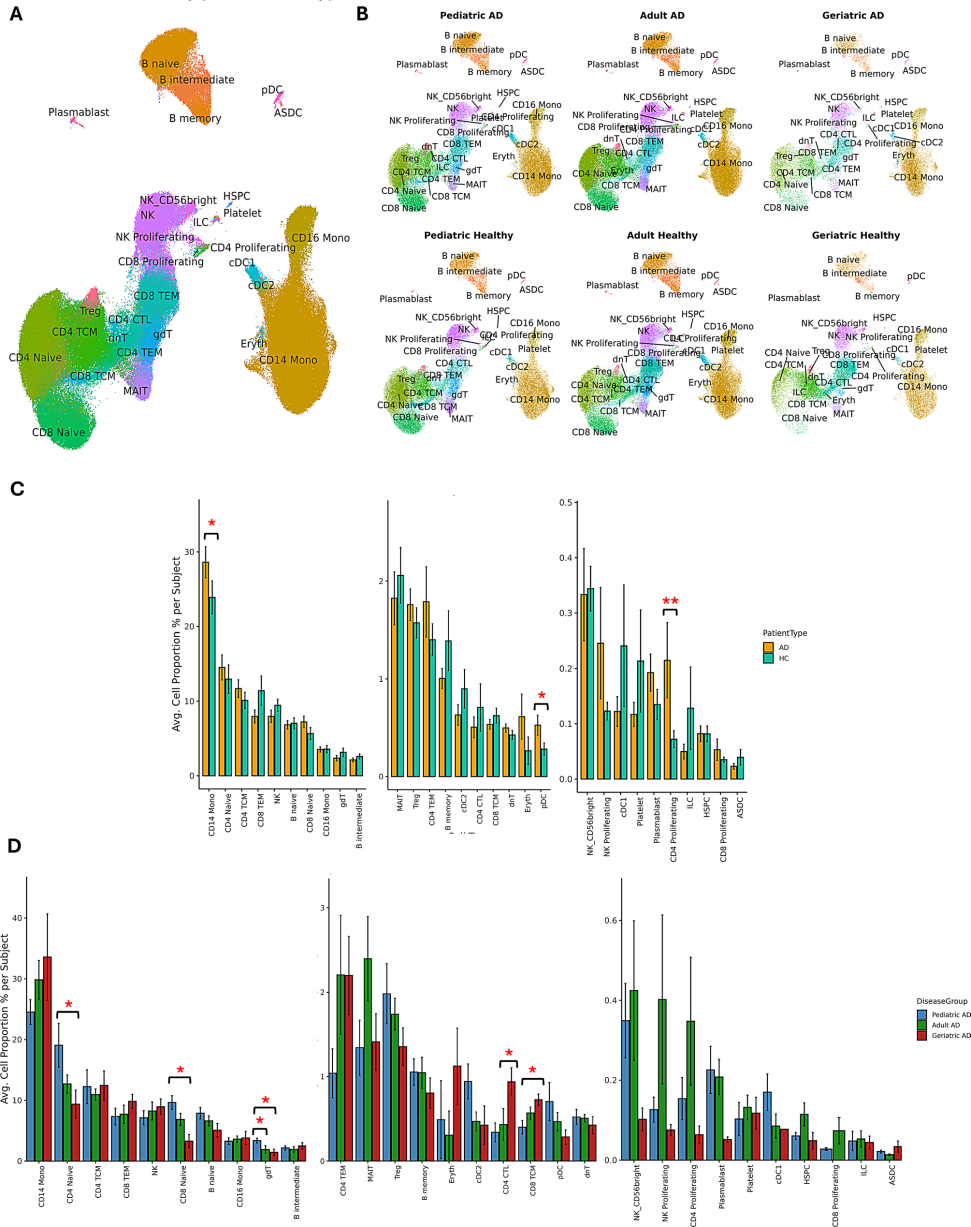
Immune landscape and cell-type composition in AD vs. HC and across AD age groups. (**A**) UMAP of all PBMCs showing 30 phenotypic subsets annotated by lineage and subset identity. (**B**) UMAPs split by patient status and age group (pediatric, adult, geriatric; AD and HC), demonstrating consistent representation of all major immune compartments across six subgroups. (**C**) Bar plots of mean cell-type proportions (AD vs HC) showing significant enrichment of CD14+ monocytes, pDcs, and CD4+ proliferating T cells (Wilcoxon, unadjusted *p* < 0.05). (**D**) Bar plots comparing AD subgroups only, highlighting significant age-linked differences in γδ T, CD4 naïve, and CD8 naïve populations (Kruskal–Wallis and pairwise Wilcoxon, *p* < 0.05)

We characterized the immune cell composition in peripheral blood mononuclear cells (PBMCs) from 29 subjects with atopic dermatitis (AD) (11 pediatric, 13 adult, and 5 geriatric) and an equal number of matched healthy controls. The CITE-seq profiling captured high-quality singlets 282,197 total cells (median ≈ 4,445) with paired quantification of 140 surface proteins. The integrated dataset spanned 30 distinct immune subsets, encompassing major lymphoid and myeloid immune cell populations. These clusters were consistently represented across all six subgroups of pediatric, adult, and geriatric AD and HC, indicating robust integration and preservation of canonical PBMC identities (Figs. 1A–B).

When cell compositions were compared between AD and HC samples, CD14+ monocytes (*p* = 0.03) and plasmacytoid dendritic cells (pDC; *p* = 0.03) were significantly increased in AD, alongside an expansion of CD4+ proliferating T cells (*p* = 0.01) (Fig. 1C). To investigate age effects within AD, we next restricted the analysis to AD patients and compared pediatric, adult, and geriatric subgroups (Fig. 1D). The Kruskal–Wallis test identified significant differences in γδ T cells (*p* = 0.01), CD8+ naïve T cells (*p* = 0.02), and CD4+ naïve T cells (*p* = 0.03). Pairwise analyses showed that pediatric AD exhibited higher cell-proportions of γδ T cells relative to both adult and geriatric groups (*p* = 0.01 for each comparison) and increased CD4+ and CD8+ naïve T cells compared to geriatric AD (*p* = 0.03 and 0.01, respectively). Conversely, CD4+ cytotoxic T cells (CD4+ CTL; *p* = 0.02) and CD8+ central memory T cells (CD8+ TCM; *p* = 0.04) were relatively elevated in geriatric AD. Together, these results reveal a developmental trajectory characterized by effector cell enrichment in AD with aging. These compositional trends prompted downstream transcriptomic and proteomic analyses to delineate the molecular programs underlying these age-linked immune shifts.

### Differential expression analysis between all AD vs all HC reveals shared downstream Th2 immune Activation

To examine overall immunological differences prior to age-group comparisons, we performed a differential expression analysis comparing all AD subjects to all age-matched HC across immune cell types, complemented by dot plots of normalized expression (Supplementary Figures 3–9, Additional File 5) and pathway enrichment analysis (Supplementary Table 3, Additional File 6). Core Th2 effector cytokine transcripts—*IL-4*, *IL-5*, and *IL-13*—were not detected as significantly differentially expressed in peripheral blood, consistent with the transient nature of cytokine transcription in circulating T cells. Supporting this, Zhang et al. demonstrated that the Th2/Th22 transcriptional signature was minimally detected in PBMCs and independent of disease severity in AD [9]. Despite this, downstream markers of Th2 immune activity were consistently elevated across multiple cell compartments. In regulatory T cells (Tregs), *CCR10* was significantly upregulated (p_adj = 6.88 × 10^−5^, log₂FC = 1.87), alongside nominal transcriptional elevation of *CCR4* (p_val = 5.45 × 10^−4^, log₂FC = 0.93) (Supplementary Figure 5A-D, Additional File 5) and independently confirmed upregulation of its surface protein CD194 (p_adj = 1.95 × 10^−4^, log₂FC = 0.19) by CITE-seq ADT (Supplementary Figure 9A-D, Additional File 5). Both *CCR10* and *CCR4* are established markers of Th2/Th22-associated immune programming [4]. In CD14+ monocytes, *JAK2* was among the most significantly upregulated genes across all AD subjects (p_adj = 3.26 × 10^−165^, log₂FC = 0.41), alongside *NOTCH2* (p_adj = 1.46 × 10^−38^, log₂FC = 0.19) (Supplementary Figure 3A-D, Additional File 5), recapitulating the Th2-priming myeloid signature described by Jin et al [3, 4]. Additionally in CD14+ monocytes, *IL13RA1* showed a nominal trend toward upregulation across all age groups (p_val = 9.86 × 10^−2^, log₂FC = 0.19) (Supplementary Figure 3A-D, Additional File 5), suggesting myeloid priming by IL-13 signaling shared across the AD cohort. Pathway enrichment analysis (Supplementary Table 3, Additional File 6) further revealed *CCR4* chemokine receptor binding in CD4+ effector memory T cells (*p* = 0.044), JAK-STAT cytokine signaling in CD4+ central memory T cells (*p* = 1.98 × 10^−3^), and *NOTCH3* activation in conventional dendritic cells type 2 (cDC2; *p* = 0.020), consistent with Th2-associated NOTCH signaling in antigen-presenting cells [4]. Taken together, these findings establish convergent evidence of an active downstream Th2 immune signature in peripheral blood across pediatric, adult, and geriatric AD, demonstrating a shared immunological baseline upon which age-specific immune programs are superimposed, as characterized in the analyses below.

### Differentially expressed genes and proteins reveal age-specific programs across AD age groups

**Fig. 2:**
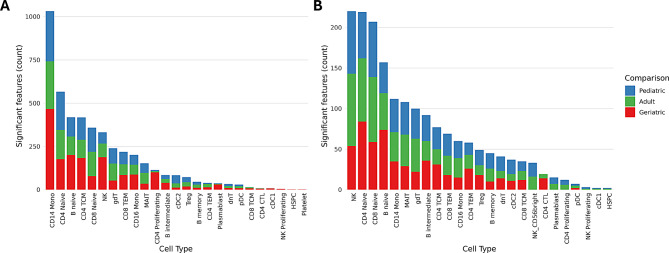
Degs and DEPs by cell type and AD subgroup comparison. (**A**) Stacked bar plot of DEG counts per cell type × comparison (pediatric AD vs other AD, PvO, adult AD vs other AD, AvO, geriatric AD vs other AD, GvO). (**B**) Stacked bar plot of DEP counts per cell type × comparison

**Fig. 3:**
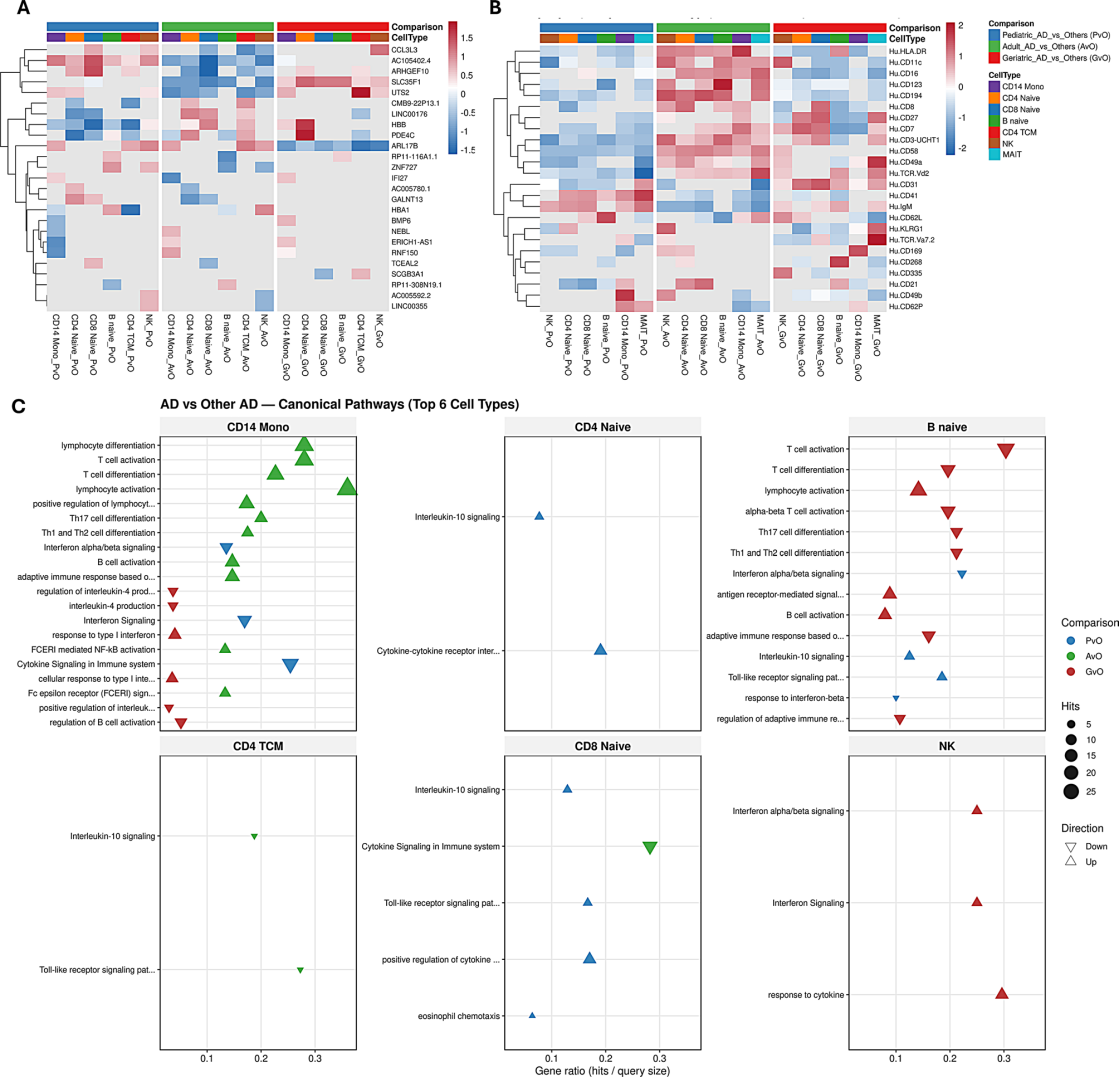
Transcriptomic and proteomic signatures reveal shared and age-specific programs across AD groups. (**A**) Heatmap of the top three “sentinel” differentially expressed genes (DEGs) per cell type and comparison—pediatric AD vs others (PvO), adult AD vs others (AvO), and geriatric AD vs others (GvO)—illustrating distinct transcriptional modules across CD14+ monocytes, CD4+ naïve, CD8+ naïve, B naïve, CD4+ TCM, and NK cells. (**B**) Heatmap of the top three “sentinel” differentially expressed proteins (DEPs) per cell type and comparison showing complementary remodeling of activation, adhesion, and immunoregulatory pathways across the same six lineages. (**C**) Pathway enrichment analysis of the full DEG set, highlighting canonical immune programs enriched by age group and cell type, including interferon/cytokine signaling, Th1/Th17 activation, chemotaxis, and cyclic adenosine monophosphate (cAMP) regulation. Collectively, these analyses reveal transcriptional and proteomic re-wiring across the AD lifespan—from interferon and cytokine activation in pediatric AD, to inflammatory and stress-responsive remodeling in adults, to metabolic and adhesion-driven adaptation in geriatric AD. Color scale represents log Fold change (logFC), where red indicates higher expression in the indicated comparison group relative to other AD groups, and blue indicates lower expression

We next compared pediatric, adult, and geriatric AD by identifying differentially expressed genes (DEGs) and proteins (DEPs) across immune cell types. Individual subsets showed 220–467 DEGs (median = 310) and 3–18 DEPs (median = 6) distinguishing age groups, with the greatest transcriptomic changes in CD14+ monocytes, followed by CD4+ naïve, CD8+ naïve, B naïve, CD4+ TCM, and NK cells (Fig. 2A). Surface protein differences were most pronounced in NK, CD4+ naïve, CD8+ naïve, B naïve, CD14+ monocyte, and mucosal-associated invariant T (MAIT) subsets (Fig. 2B), reflecting partially overlapping but distinct age-associated patterns. To highlight representative signatures, the top three DEGs or DEPs per cell type and age-group contrast (AD vs. other AD groups) were selected, filtering out features with limited detection (fewer than two cell type–comparison combinations) or lacking variability in log₂ fold-change to ensure robust clustering across prioritized lineages (Figs. 3A–3B).

At the transcriptomic level, pediatric AD showed increased expression of transcription factor and signaling genes (e.g., *IFI27, ZNF727, ARHGEF10*) and trafficking or structural regulator *GALNT13*. The long noncoding RNA (lncRNA) *AC105402.4* was uniquely upregulated, suggesting age-specific epigenetic control. In adult AD, these same genes were downregulated, while differentiation- and metabolic-stress-related transcripts (e.g., *ERICH1–AS1, LINC00176, NEBL*) were elevated. Geriatric AD exhibited elevated signaling and metabolic regulators (e.g., *IFI27, BMP6, PDE4C, SLC35F1, HBB*) and persistent *ERICH1–AS1* expression, with unique downregulation of *ARL17B*.

Pathway analysis of the full DEG set (Fig. 3C) showed enrichment of IL-10 and cytokine–cytokine receptor signaling in pediatric AD naïve compartments (B Naïve, CD4+ Naïve T, and CD8+ Naïve T). In terms of innate activation, toll-like receptor (TLR) activity was reduced specifically in CD14+ monocytes, whereas it was upregulated in pediatric B Naïve and CD8+ Naïve T cells. In contrast, adult AD was enriched for monocyte-driven Th1/Th17/NF-κB activation and B-cell stimulation. Geriatric AD retained type-1 interferon activity and IL-10 signaling in monocytes and B cells but displayed reduced IL-4 production and T-cell signaling, while NK cells demonstrated elevated interferon and cytokine responses, reflecting a shift toward innate immune activation with aging.

Surface protein profiling revealed complementary remodeling across programs involving T-cell activation, trafficking, B-cell signaling, and innate regulation (Fig. 3B). Pediatric AD showed upregulation of B-cell and immunoglobulin (B/Ig)-related proteins (e.g., IgM, CD41, CD62L) and downregulation of trafficking or naïve-lineage receptors (e.g., CD49a, CD8), suggesting early B-cell activity and a predominance of maturing lymphocytes. Adult AD exhibited stronger T-cell activation and differentiation, marked by increased activation, trafficking, and checkpoint proteins (HLA-DR, CD49a, CD21, KLRG1, CD16), consistent with enhanced effector and regulatory responses. Geriatric AD displayed upregulation of co-stimulatory and adhesion molecules (CD27, CD31, CD49a), together with myeloid and NK-associated markers (CD169, CD268, CD335) and unconventional T-cell receptors (TCR Vδ2, Vα7.2), reflecting adaptive and innate immune adjustments with aging.

### Machine learning classifiers distinguish pediatric, adult, and geriatric AD

We next evaluated the capacity of gene- and protein-level features to distinguish pediatric, adult, and geriatric AD. Using AD-only input matrices derived from significant DEGs and DEPs, we trained multi-class machine learning models to test whether compact molecular signatures could classify participants by age group. Model development used nested internal cross-validation for feature selection and hyperparameter tuning within the training set, followed by a single 40% held-out validation set for performance estimation. Due to the moderate sample size, particularly in the geriatric cohort, metrics reflect validation performance rather than full cross-subject cross-validation. Regularization and inverse-frequency class weighting were applied across all algorithms to control model complexity and mitigate class imbalance.

**Fig. 4:**
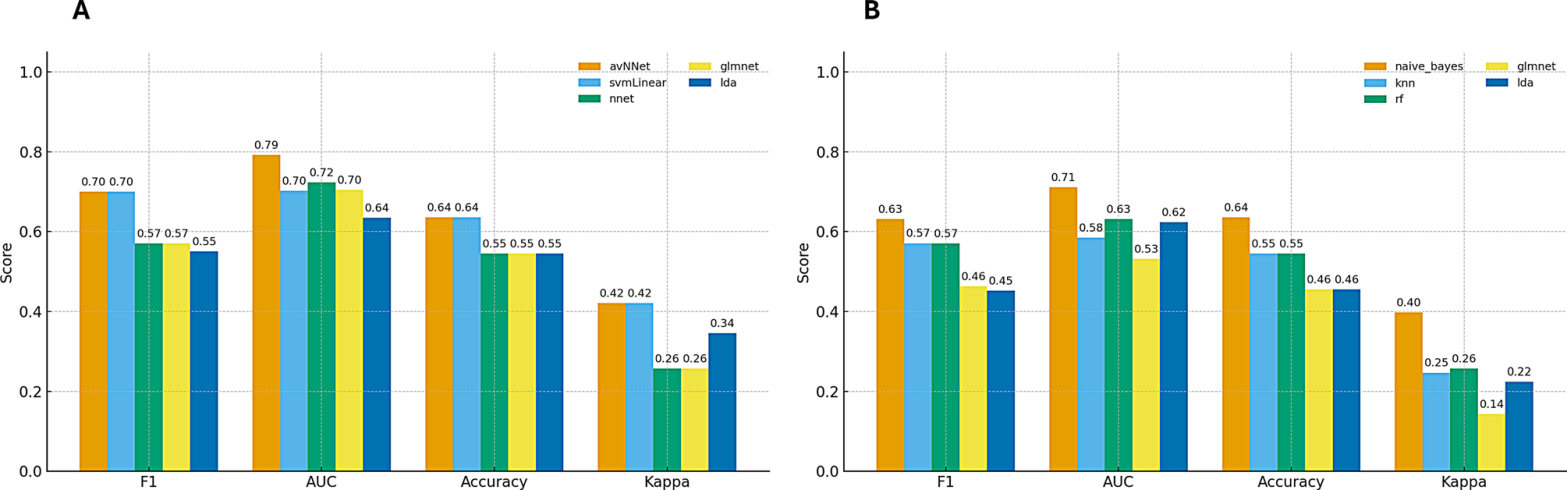
Performance of the top 5 machine learning classifiers. (**A**) Classifiers trained on DEG-based feature sets. (**B**) Classifiers trained on DEP-based feature sets

**Fig. 5:**
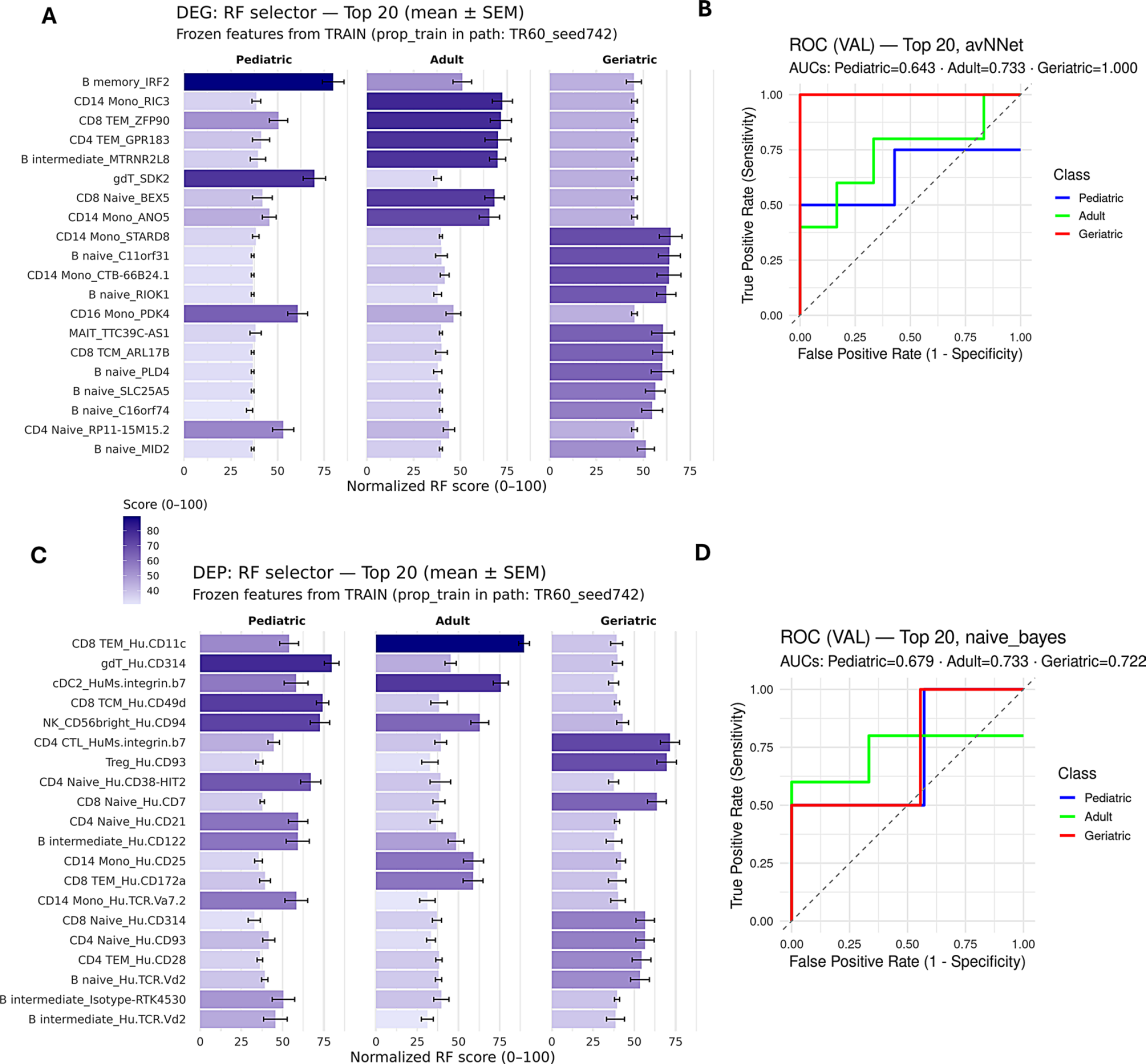
Feature importance across gene and protein modalities and validation model performance. (**A**) Top 20, stable gene features ranked by random forest importance. (**B**) Receiver operating characteristic (ROC) curves of the best-performing gene-based model (avNnet). (**C**) Top 20, stable protein features ranked by random forest importance. (**D**) ROC curves of the best-performing protein-based model (naïve Bayes)

Across eight machine learning algorithms, DEG-based classifiers consistently outperformed DEP-based models. The avNNet and SVM Linear models yielded the highest validation performance, with avNNet reaching F1 = 0.70, AUC = 0.79, and accuracy = 0.64, while SVM Linear achieved AUC = 0.70 (Fig. 4A). In contrast, DEP-based models demonstrated moderate discrimination, with Naive Bayes performing best (macro F1 ≈ 0.63, AUC ≈0.71, accuracy ≈0.64) (Fig. 4B). The geriatric AUC of 1.0 (Fig. 5B) observed in the DEG validation set likely reflects data sparsity—only two geriatric samples in the validation set and three in the training set—rather than true perfect classification.

The top 20 stable DEGs (Fig. 5A) identified through Random Forest feature selection encompassed transcriptional and signaling regulators (*IRF2, ZFP90, GPR183, RIC3*), structural and trafficking mediators (*SDK2, STARD8, ANO5, MID2*), and epigenetic or lncRNA-associated genes (*CTB-66B24.1, RP11-15M15.2, ARL17B, TTC39C-AS1*). Additional features included protein synthesis and ubiquitin-related genes (*RIOK1, SLC25A5*) and metabolic regulators (*MTRNR2L8, PDK4*) (Fig. 5A). In parallel, the top 20 DEPs identified through Random Forest feature selection (Fig. 5C) encompassed T-cell activation and differentiation markers (CD28, CD25, CD38–HIT2), trafficking and homing receptors (CD49d, integrin β7, CD172a, CD11c), NK-related proteins (CD94, CD314), and B-cell or immunoglobulin-associated molecules (CD21, CD122). Additional γδ/MAIT T-cell receptors (TCR Vδ2, TCR Vα7.2) and regulatory proteins (CD93) reflected the integrated remodeling of adaptive and innate immune programs across age groups.

Collectively, these results demonstrate that compact, biologically interpretable feature sets derived from differential expression are sufficient to separate AD age groups with moderate accuracy, supporting their utility as age-stratified molecular signatures for downstream biological interpretation.

### Biological interpretation of stable gene and protein features across AD age groups

**Fig. 6:**
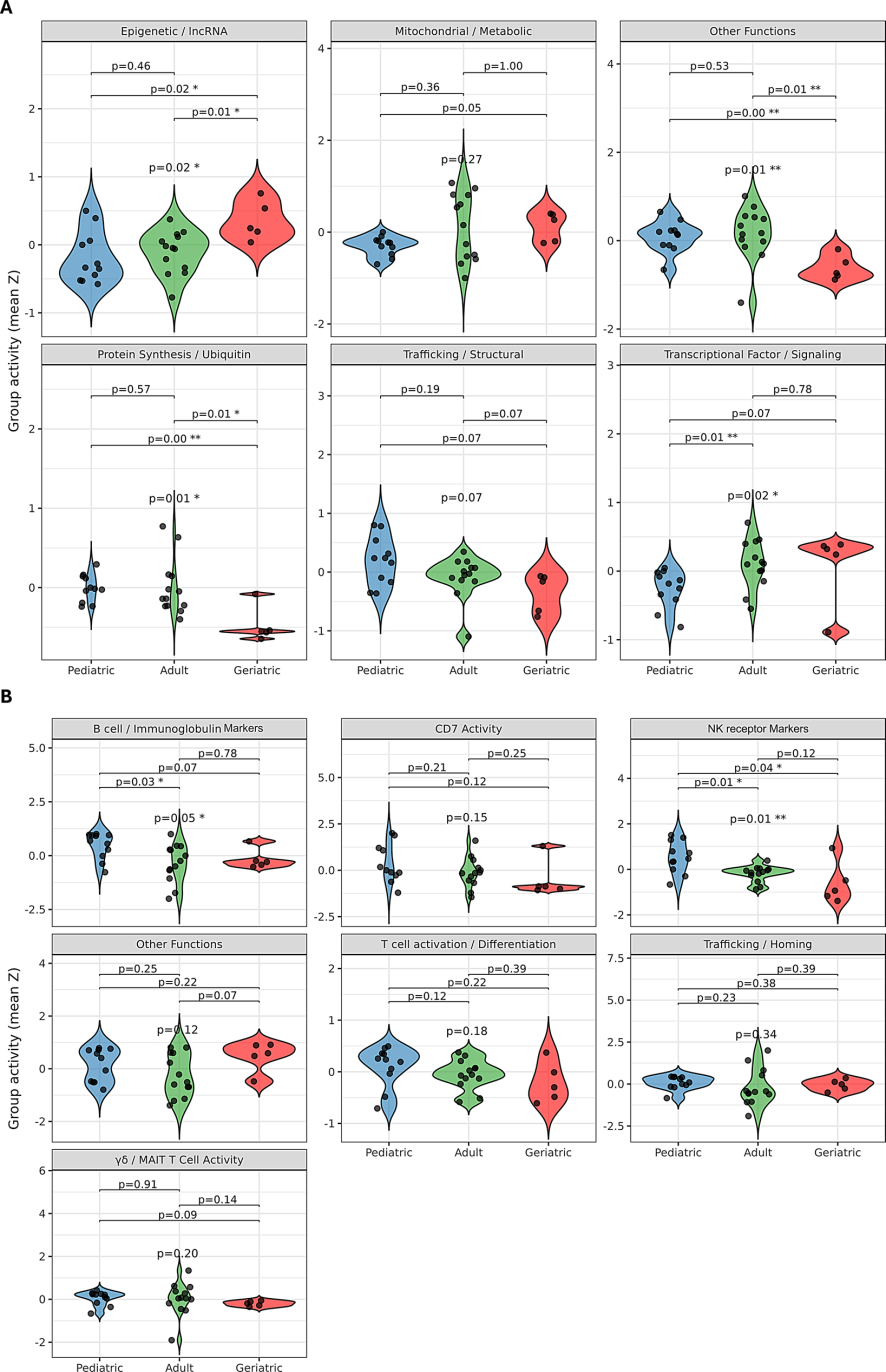
Functional group activity across stable genes and proteins. (**A**) Violin plots showing mean Z-scored expression of the top 20 stable genes grouped by functional activity. (**B**) Violin plots showing mean Z-scored expression of the top 20, stable proteins grouped by functional activity

We next examined the biological relevance of these stable DEGs and DEPs. These features collectively captured transcriptional, structural, and metabolic programs that differentiated pediatric, adult, and geriatric AD while maintaining cell-type diversity across B cells, monocytes, and T-cell lineages (Fig. 6A–B). The Z-scored expression of the stable top 20 gene and protein features is detailed in heatmaps in Supplementary Figures 10A-B, Additional File 7, illustrating the consistent, age-dependent transcriptional and proteomic remodeling that we subsequently analyzed by functional group (Supplementary Tables 4–5, Additional Files 8–9).

Significant age-associated variation was observed in transcriptional signaling, protein synthesis/ubiquitin, and epigenetic/lncRNA regulation (*p* = 0.02, 0.01, and 0.02, respectively; Fig. 6A). While pediatric AD exhibited modest, non-significant higher expression in structural and trafficking genes, these patterns are consistent with early cytoskeletal and translational programs characteristic of the pediatric age-specific overlay [10]. In contrast, adult AD demonstrated significantly higher expression of transcriptional regulators (*IRF2, ZFP90*) and metabolic mediators (*PDK4, RIC3*) compared to pediatric AD (*p* = 0.01), reflecting peak transcriptional and metabolic remodeling as the dominant adult-specific overlay. Geriatric AD, by comparison, displayed significantly lower expression of protein synthesis/ubiquitin (*RIOK1, SLC25A5*; *p* = 0.01 compared to pediatric AD and *p* = 0.01 compared to adult AD) and lncRNA-associated genes (*RP11-15M15.2*; *p* = 0.02 compared to pediatric AD and *p* = 0.01 compared to adult AD), consistent with diminished translational activity and a compensatory shift toward innate immunity, a characteristic of inflammaging [11]. Collectively, these findings support age-specific molecular overlays superimposed on a shared Th2 baseline: pediatric AD defined by early structural and regulatory programs, adult AD by peak transcriptional and metabolic remodeling, and geriatric AD by inflammaging.

At the protein level, significant age-associated differences were observed in NK receptor markers (*p* = 0.01) and B cell/Immunoglobulin receptor markers (*p* = 0.05) (Fig. 6B). Both groups showed the highest expression in pediatric AD, with lower levels in adult (*p* = 0.01, 0.03, respectively) and geriatric (*p* = 0.04) groups, indicating stronger NK- and B-cell receptor marker expression in early disease that diminishes with age. Key proteins driving these differences included NK-related CD94 and CD314, and B/Ig-related CD21 and CD122. In contrast, T-cell activation and differentiation markers (CD28, CD25, CD38–HIT2), trafficking and homing proteins (CD49d, integrin β7, CD172a, CD11c), and γδ/MAIT T-cell receptors (TCR Vδ2, TCR Vα7.2) remained relatively stable across age groups, consistent with the shared Th2 effector underlying the AD lifespan [3]. Together, these findings highlight that NK and B-cell receptor marker expression constitutes an age-stratified overlay. In contrast, the stability of T-cell and trafficking receptor markers reflects the common Th2 baseline across pediatric, adult, and geriatric AD.

## Discussion

Our study sheds light on immune composition differences among pediatric, adult, and geriatric patients with AD at multiple levels of resolution, from PBMCs. At the cell composition level, AD patients showed higher proportions of CD14+ monocytes and pDC compared with HC, consistent with earlier reports of altered dendritic-cell balance in AD [4, 12]. Differential expression analysis across all AD subjects relative to HC further revealed convergent downstream signatures of Th2-associated immune activity shared across all three age groups: upregulation of *CCR10* and *CCR4*/CD194 in Treg cells, *JAK2* and *NOTCH2* in CD14+ monocytes, and enrichment of JAK-STAT and *NOTCH3* signaling pathways [3, 4]. These downstream activities establish that Th2 constitutes the shared immunological baseline across the AD lifespan.

Superimposed on this foundation, age-stratified analyses revealed distinct immune programs within the AD cohort. Pediatric patients had more γδ T cells and naïve CD4+ and CD8+ T cells than adults or older patients, suggesting a less mature immune profile with greater naïve T-cell proportions early in life [13]. In contrast, geriatric AD was marked by an increase in CD4+ CTL and CD8+ TCM, reflecting a shift toward more mature and effector cell types. Together, these changes suggest that AD follows and exacerbates the general pattern of immune aging, characterized by reduced naïve and increased effector and memory populations [11, 14]. Our subsequent analyses characterize this age-specific exacerbation in detail, with geriatric AD representing an inflammaging overlay superimposed on the shared Th2-driven baseline of AD. The signatures reported subsequently represent this age-stratified molecular change: transcriptional and proteomic features that distinguish pediatric, adult, and geriatric AD from one another, rather than markers of AD relative to healthy controls, and should be interpreted accordingly.

Within the most variable immune cell types (CD14+ monocytes, CD4+ naïve, CD8+ naïve, B naïve, CD4+ TCM, and NK cells), we observed progressive age-linked remodeling of immune programs in AD (Fig. 3). In pediatric AD, upregulation of transcriptional and signaling regulators (*IFI27, ZNF727, ARHGEF10*) and the structural gene (*GALNT13*), together with the lncRNA *AC105402.4*, indicates coordinated activation of regulatory and structural pathways that overlap with developmental and differentiation programs. A prominent feature of the pediatric AD landscape is the enrichment of IL-10 signaling in naïve T- and B-cell compartments—a regulatory program consistent with IL-10‘s established role in allergen tolerance and immune modulation [15], and with the immune plasticity that defines early-life responses to chronic antigenic exposure [16]. This IL-10 signature co-exists with the shared Th2-driven inflammatory baseline, tempering the broader type 2 response during a period when immune identity is still being shaped [15]. The concurrent reduction of toll-like receptor signaling in CD14+ monocytes but increased responsiveness in naïve B and T cells further supports selective restraint of innate pathways alongside heightened adaptive priming, consistent with this early-life immune plasticity [16]. In adult AD, the downregulation of early transcriptional regulators (*IFI27, ZNF727, ARHGEF10*) and upregulation of stress-response and non-coding regulatory genes (*ERICH1–AS1, HBB, PDE4C, LINC00176, NEBL*) reflect a transcriptional program distinct from the developmental signatures observed in pediatric AD. Pathway enrichment for Th2, Th1, Th17, and NF-κB signaling, particularly within CD14+ monocytes, indicates broad effector remodeling consistent with reports that adult AD exhibits stronger Th1/Th17 activity than pediatric AD [3]. The concurrent decrease of IL-10 and TLR signaling in CD4+ central-memory T cells reflects diminishment of the tolerance network and the emergence of an effector-dominant inflammatory state. In geriatric AD, persistent interferon and IL-10 signaling accompanied higher expression of metabolic and mitochondrial regulators (*BMP6, SLC35F1*), suggesting adaptive, energy-conserving responses typical of immune aging [11, 17]. Notably, *ERICH1–AS1, HBB*, and *PDE4C*, which are stress-response and metabolic genes shared with the adult AD, were retained in the geriatric transcriptional program, suggesting that geriatric AD inherits core adult inflammatory programs while overlaying aging-specific innate and regulatory features [11, 17]. Reduced IL-4 production and T-cell signaling, together with transcriptional enrichment of interferon and cytokine pathways in geriatric NK cells (Fig. 3C), highlight a shift toward innate immune compensation. Notably, while NK-associated receptor surface expression was lower in geriatric AD relative to younger age groups (Fig. 6B), this pattern reflects reduced cytotoxic receptor availability consistent with immunosenescence rather than global NK inactivity—a dissociation between cytotoxic capacity and inflammatory signaling previously described in severe AD [4]. The presence of adhesion and co-stimulatory genes (*BMP6, SLC35F1*) and increased myeloid-associated proteins (CD169, CD268, CD335) is consistent with an inflammaging overlay characterized by chronic low-grade inflammation coupled with compensatory regulatory signaling within the aging immune network.[4, 11].

Surface-protein analyses supported these transcriptomic trends. In pediatric AD, upregulation of B-cell and immunoglobulin-related proteins (IgM, CD41, CD62L) and downregulation of trafficking and γδ T-cell receptor markers (CD49a, TCR Vδ2) indicate early B-cell activity and a predominance of maturing lymphocytes. While enrichment of naïve and transitional B-cell subsets has been reported in adult AD blood [18], our findings provide a detailed proteomic profile of circulating B cells in pediatric AD, identifying specific surface markers of B-cell maturation that characterize early disease development. In adult AD, elevated expression of activation, trafficking, and checkpoint proteins marks the peak of chronic immune activation. Increased antigen presentation (HLA-DR), T-cell trafficking (CD49a), B-cell activation (CD21), and cytotoxic and effector activity (KLRG1, CD16) reflect heightened engagement of both adaptive and innate pathways characteristic of sustained inflammatory remodeling [4, 13, 18]. This profile aligns with our transcriptomic findings of enhanced Th1/Th17 and NF-κB signaling, reinforcing that adult AD represents a state of maximal immune activation and metabolic stress. In geriatric AD, upregulation of co-stimulatory and adhesion molecules (CD27, CD31), myeloid and NK-associated proteins (CD169, CD268, CD335) was observed, consistent with an inflammaging phenotype [11]. Notably, CD27 expression was increased in both adult and geriatric AD, consistent with prior reports of expanded CD27+ chronically activated memory B cells in AD blood [18]. CD27 is a co-stimulatory receptor expressed on activated and memory lymphocytes that promotes cell survival and sustained immune activation upon engagement with its ligand CD70. Its elevation across both age groups, together with comparable B-cell and immunoglobulin activity in adult and geriatric AD (Fig. 6B), points to a shared humoral activation state maintained across the later AD lifespan, consistent with the high prevalence of IgE sensitization and Th2-driven humoral engagement reported in both adult and geriatric AD populations.[19].

Machine learning–based feature selection and average expression analyses revealed compact, biologically coherent gene and protein signatures that distinguish pediatric, adult, and geriatric AD with moderate accuracy. Transcript-level classifiers (avNNet, SVM Linear) achieved superior performance (F1 ≈ 0.70) compared to protein-based classifiers (F1 ≈ 0.63), reflecting greater transcriptomic stability across individuals. In pediatric AD, at the transcriptomic level, *IRF2*, which encodes an interferon regulatory factor with established genetic associations with AD susceptibility, was the top-ranked DEG feature in pediatric B memory cells [20]. *IRF2* acts as a transcriptional repressor of type I IFN signaling and is required for NK cell development and B cell maturation [21, 22]; its importance in the pediatric signature may reflect heightened IFN-regulatory activity within the maturing B cell compartment, consistent with early immune programming under chronic allergen exposure. Complementary to this lymphoid signal, *PDK4*, which encodes pyruvate dehydrogenase kinase 4, a metabolic checkpoint that suppresses mitochondrial pyruvate oxidation and redirects carbon flux toward aerobic glycolysis, was a top-ranked feature in pediatric CD14+ monocytes [23]. In other contexts, *PDK4*-driven aerobic glycolysis has been associated with M1-like pro-inflammatory cytokine production in myeloid cells [23]; its importance suggests that innate myeloid metabolic activation is an early feature of pediatric AD, occurring alongside the regulatory and developmental immune programs that characterize this age group. At the proteomic level, key ML features such as CD21, CD94, and CD122 align with the early B-cell and NK-cell activation observed in the differential protein analysis. CD94, a C-type lectin receptor that regulates NK cell activation thresholds through inhibitory and activating NKG2 heterodimer pairing, supports a state of heightened NK cell readiness consistent with prior reports of NK cell receptor dysregulation in pediatric AD [24]. In adult AD, the strong weighting of *RIC3*, *ZFP90*, *GPR183*, *MTRNR2L8*, *BEX5*, and *ANO5* suggests peak transcriptional and metabolic remodeling, consistent with the DEG findings, which capture the convergence of adaptive and metabolic stress responses typical of chronic inflammation. Among these, *GPR183* (EBI2) encodes an oxysterol-sensing chemotactic receptor that regulates effector memory T cell positioning and trafficking [25]; its upregulation in CD4+ effector memory T cells is consistent with enhanced lymphocyte trafficking under the lipid-dysregulated microenvironment of chronic AD. *RIC3*, a chaperone required for surface expression of the α7 nicotinic acetylcholine receptor in CD14+ monocytes, raises the possibility that the cholinergic anti-inflammatory pathway may be engaged as a counterregulatory response to the pro-inflammatory myeloid activation captured by concurrent *PDK4* upregulation—though whether these programs interact in the AD monocyte compartment remains to be determined [26]. Together, these features capture the convergence of adaptive trafficking, metabolic stress, and innate immune modulation characteristic of chronic adult AD. In geriatric AD, for the DEG classifier, *STARD8*, a RhoGAP and lipid-transfer domain protein, was the top-ranked DEG feature in geriatric CD14+ monocytes, consistent with myeloid lipid metabolic dysregulation associated with inflammaging and the known role of *STARD8*/*DLC3* as a RhoGAP in cytoskeletal regulation [27, 28]. For the DEP classifier, integrin β7 and CD7 are both top-ranked features, reflecting enhanced tissue-homing capacity and innate-like T cell conversion consistent with inflammaging. As with the features described across all three age groups, these interpretations are informed by the known biology of these features in other contexts and should be considered hypothesis-generating, pending functional validation in AD-specific experimental models. When aligned with the transcriptomic and surface-protein differential expression and pathway analyses, these ML-derived features delineate a coherent age trajectory superimposed on a Th2 baseline: pediatric AD marked by early regulatory and developmental programs (IL-10, IFN signaling, B cell + NK priming), adult AD peak transcriptional and metabolic remodeling (Th1/Th17, NF-κB), and geriatric AD by inflammaging (innate compensation, compensatory co-stimulation, reduced adaptive dominance). Together, these patterns demonstrate that compact, cell-informed molecular signatures can capture the immunologic progression from developmental immune readiness to chronic inflammatory remodeling and age-related innate compensation in AD.

There were several limitations to this study. Sample collection was cross-sectional and did not track patients longitudinally, preventing direct assessment of temporal immune shifts. However, we minimized confounding by excluding subjects receiving systemic immunomodulators at the time of sampling. The moderate overall sample size limited model generalizability and constrained feature selection in machine-learning analyses. In particular, the geriatric AD classifier’s AUC should be interpreted cautiously given the very limited geriatric validation sample size (*n* = 2); the observed performance should be interpreted in the context of a small sample size and may be susceptible to overfitting. Additionally, our analysis was restricted to circulating immune cells, which may not fully capture skin-resident or tissue-specific activity. Furthermore, subjects were not characterized by IgE sensitization status (extrinsic [IgE-allergic] versus intrinsic [non-allergic] AD phenotype); prior clinical studies have demonstrated heterogeneity in IgE profiles among AD patients, suggesting distinct immunologic subtypes, while molecular analyses have shown that intrinsic AD exhibits similar Th2 but increased Th17 immune activation compared to extrinsic AD [29, 30]. The presence or absence of IgE sensitization may therefore influence the cytokine and immune signatures observed across age groups. Finally, while the multi-omics signatures identified here are supported by established biological precedents for key features, direct functional and clinical validation of these age-specific programs remains an important next step and a recognized limitation of this discovery-phase study. Future studies integrating matched skin–blood single-cell data, longitudinal sampling, and perturbation experiments will be essential to validate these pathways and evaluate therapeutic relevance. Although clinical outcome measures were not directly incorporated in this analysis, the identified pathways align with established disease-relevant immune mechanisms in AD and provide a foundation for future studies linking molecular signatures with disease severity, treatment response, and patient stratification.

## Conclusion

Through single-cell multi-omics profiling, we identified distinct immunologic differences across the atopic dermatitis lifespan. These age-linked immune programs yielded moderately stable molecular signatures capable of distinguishing AD subgroups, providing a foundation for age-group subtyping, prognostic refinement, and tailored therapeutic strategies for atopic dermatitis across the human lifespan.

## Electronic supplementary material

Below is the link to the electronic supplementary material.

Supplementary Material 1

Supplementary Material 2

Supplementary Material 3

Supplementary Material 4

Supplementary Material 5

Supplementary Material 6

Supplementary Material 7

Supplementary Material 8

Supplementary Material 9

Supplementary Material 10

## Data Availability

The data supporting this study’s conclusions are included within the article and its supplementary materials. Raw sequencing data have been deposited in the NCBI Gene Expression Omnibus under accession number GSE332623. Additional datasets are available from the corresponding author upon reasonable request.

## Abbreviations

ACD: Acid citrate dextrose
AD: Atopic dermatitis
ADT: Antibody-derived tag
AUC: Area under the receiver operating characteristic curve
AUROC: Area under the receiver operating characteristic curve (multi-class context)
cAMP: Cyclic adenosine monophosphate
CITE-seq: Cellular Indexing of Transcriptomes and Epitopes by Sequencing
CTL: Cytotoxic T lymphocyte
DEG(s): Differentially expressed gene(s)
DEP(s): Differentially expressed protein(s)
DMSO: Dimethyl sulfoxide
FBS: Fetal bovine serum
FDR: False discovery rate
GRCh37/GRCh38: Genome Reference Consortium Human build 37/38
HC: Healthy control
IRB: Institutional Review Board
JAK–STAT: Janus kinase–signal transducer and activator of transcription
MAIT: Mucosal-associated invariant T cell
NF-κB: Nuclear factor kappa-light-chain-enhancer of activated B cells
NK: Natural killer
PBMCs: Peripheral blood mononuclear cells
PCA: Principal component analysis
QC: Quality control
RNA: Ribonucleic acid
ROC: Receiver operating characteristic
SEM: Standard error of the mean
SNN: Shared nearest neighbor
SNP: Single-nucleotide polymorphism
TCM: Central memory T cell
Th1/Th2/Th17/Th22: T helper 1/2/17/22
TLR: Toll-like receptor
UMAP: Uniform Manifold Approximation and Projection
VCF: Variant call format
vIGA-AD: Validated Investigator Global Assessment for Atopic Dermatitis
