# Supplementary material for: Immunological differences in atopic dermatitis across age groups: insights from single-cell multi-omics
Source: J Transl Med. 2026 Jun 1;24:1004. doi: 10.1186/s12967-026-08288-7 (PMC13445865; doi:10.1186/s12967-026-08288-7)
Supplement: Supplementary file 1 [file 12967_2026_8288_MOESM3_ESM.pdf]

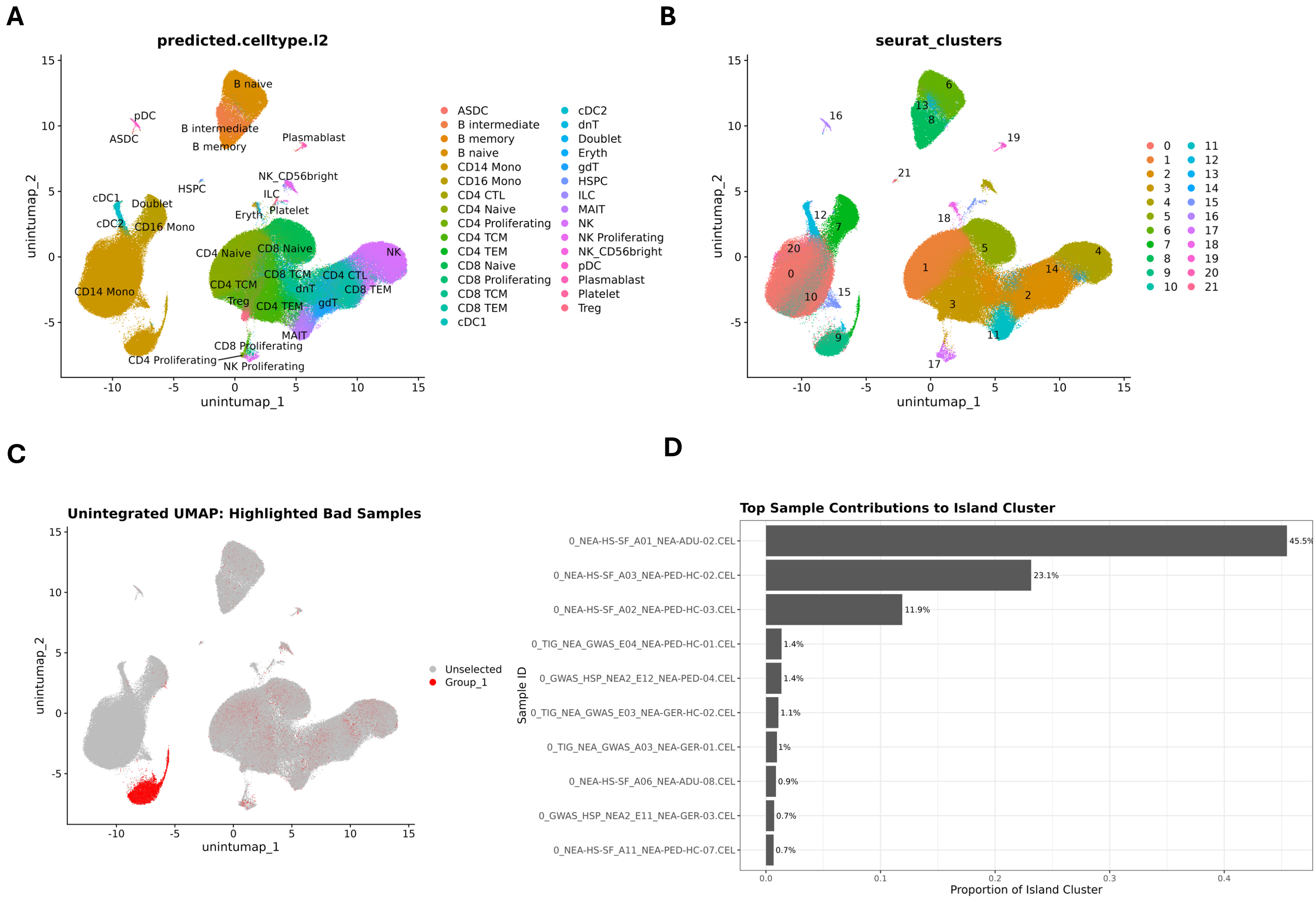
A
predicted.celltype.l2
B naive
pDC
B intermediate
ASDC
B memory
Plasmablast
NK_CD56bright
HSPC
ILC
cDC1
Doublet
Platelet
Eryth
cDC2
CD16 Mono
CD4 Naive
CD8 Naive
NK
CD8 TCM
CD4 CTL
CD4 TCM
dnT
CD8 TEM
Treg
CD4 TEM
gdT
CD14 Mono
MAIT
CD8 Proliferating
CD4 Proliferating
NK Proliferating
unintumap_1
unintumap_2
ASDC
B intermediate
B memory
B naive
CD14 Mono
CD16 Mono
CD4 CTL
CD4 Naive
CD4 Proliferating
CD4 TCM
CD4 TEM
CD8 Naive
CD8 Proliferating
CD8 TCM
CD8 TEM
cDC1
cDC2
dnT
Doublet
Eryth
gdT
HSPC
ILC
MAIT
NK
NK Proliferating
NK_CD56bright
pDC
Plasmablast
Platelet
Treg
B
seurat_clusters
unintumap_1
unintumap_2
C
Unintegrated UMAP: Highlighted Bad Samples
unintumap_1
unintumap_2
Unselected
Group_1
D
Top Sample Contributions to Island Cluster
Sample ID
Proportion of Island Cluster
0_NEA-HS-SF_A01_NEA-ADU-02.CEL 45.5%
0_NEA-HS-SF_A03_NEA-PED-HC-02.CEL 23.1%
0_NEA-HS-SF_A02_NEA-PED-HC-03.CEL 11.9%
0_TIG_NEA_GWAS_E04_NEA-PED-HC-01.CEL 1.4%
0_GWAS_HSP_NEA2_E12_NEA-PED-04.CEL 1.4%
0_TIG_NEA_GWAS_E03_NEA-GER-HC-02.CEL 1.1%
0_TIG_NEA_GWAS_A03_NEA-GER-01.CEL 1%
0_NEA-HS-SF_A06_NEA-ADU-08.CEL 0.9%
0_GWAS_HSP_NEA2_E11_NEA-GER-03.CEL 0.7%
0_NEA-HS-SF_A11_NEA-PED-HC-07.CEL 0.7%
