# Supplementary material for: Immunological differences in atopic dermatitis across age groups: insights from single-cell multi-omics
Source: J Transl Med. 2026 Jun 1;24:1004. doi: 10.1186/s12967-026-08288-7 (PMC13445865; doi:10.1186/s12967-026-08288-7)
Supplement: Supplementary file 2 [file 12967_2026_8288_MOESM4_ESM.pdf]

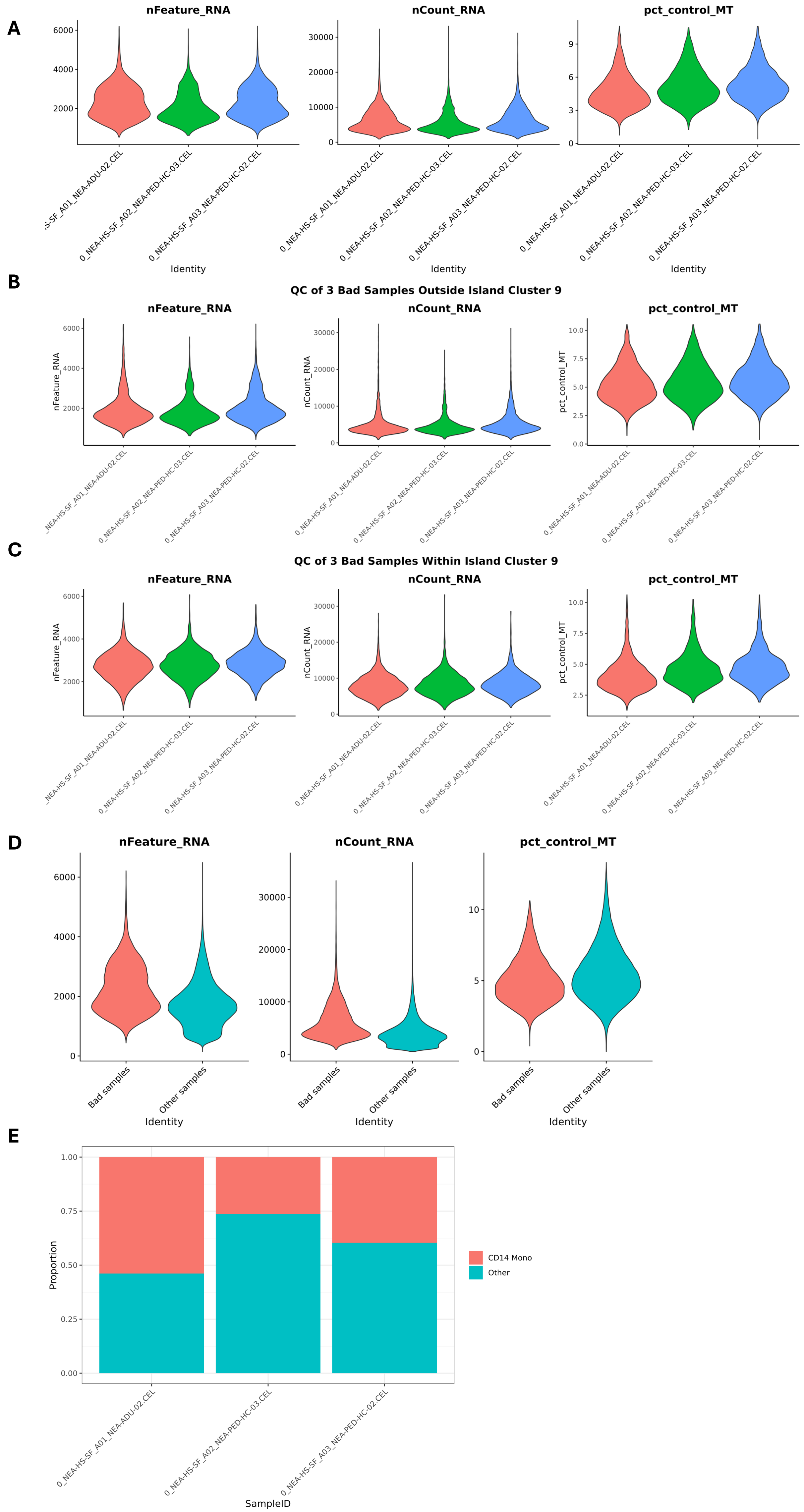
A
nFeature_RNA
nCount_RNA
pct_control_MT
0_NEA-HS-SF_A01_NEA-ADU-02.CEL
0_NEA-HS-SF_A02_NEA-PED-HC-03.CEL
0_NEA-HS-SF_A03_NEA-PED-HC-02.CEL
Identity
B
QC of 3 Bad Samples Outside Island Cluster 9
nFeature_RNA
nCount_RNA
pct_control_MT
C
QC of 3 Bad Samples Within Island Cluster 9
nFeature_RNA
nCount_RNA
pct_control_MT
D
nFeature_RNA
nCount_RNA
pct_control_MT
Bad samples
Other samples
Identity
E
Proportion
SampleID
CD14 Mono
Other
