# Supplementary material for: Immunological differences in atopic dermatitis across age groups: insights from single-cell multi-omics
Source: J Transl Med. 2026 Jun 1;24:1004. doi: 10.1186/s12967-026-08288-7 (PMC13445865; doi:10.1186/s12967-026-08288-7)
Supplement: Supplementary file 3 [file 12967_2026_8288_MOESM5_ESM.pdf]

A

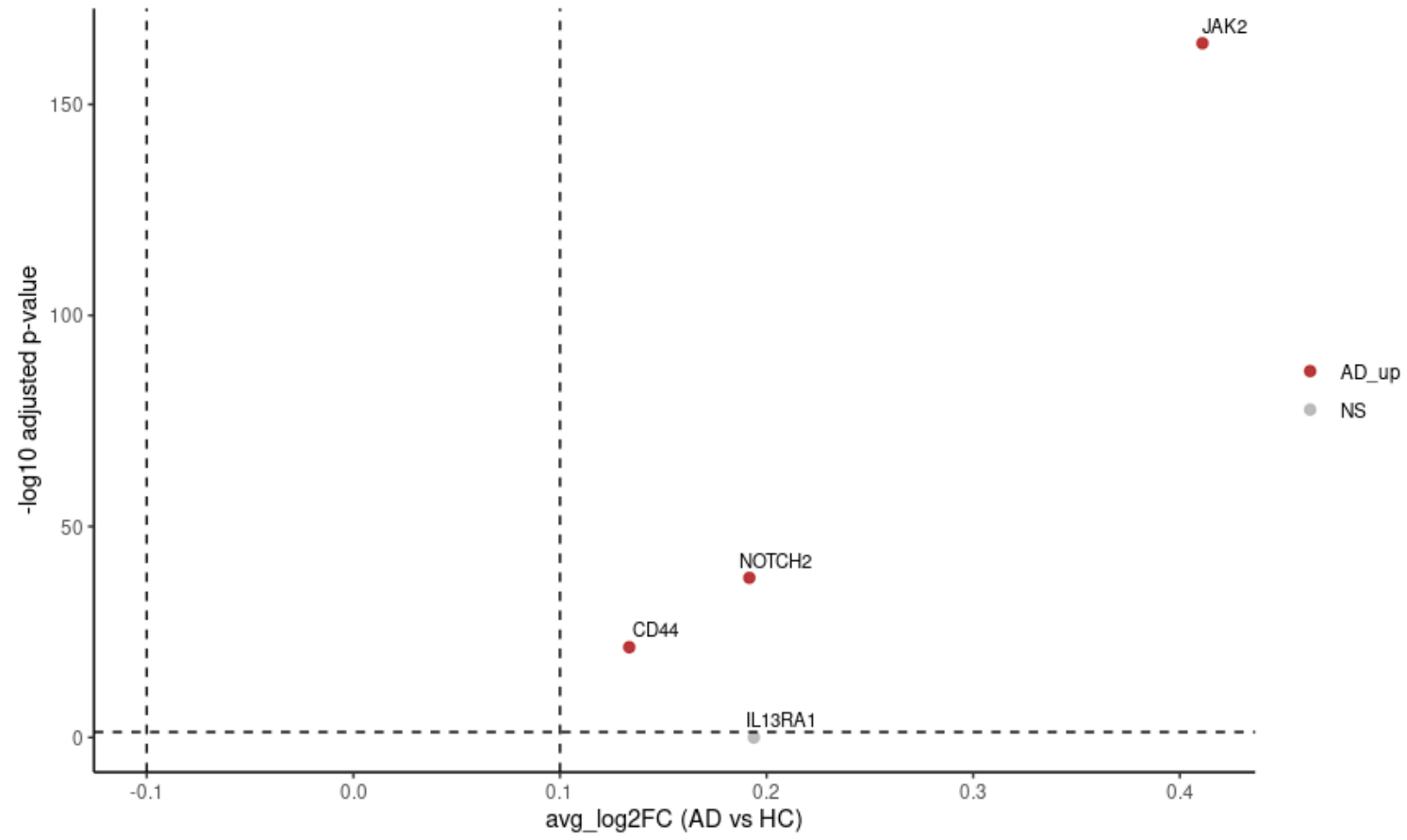

B

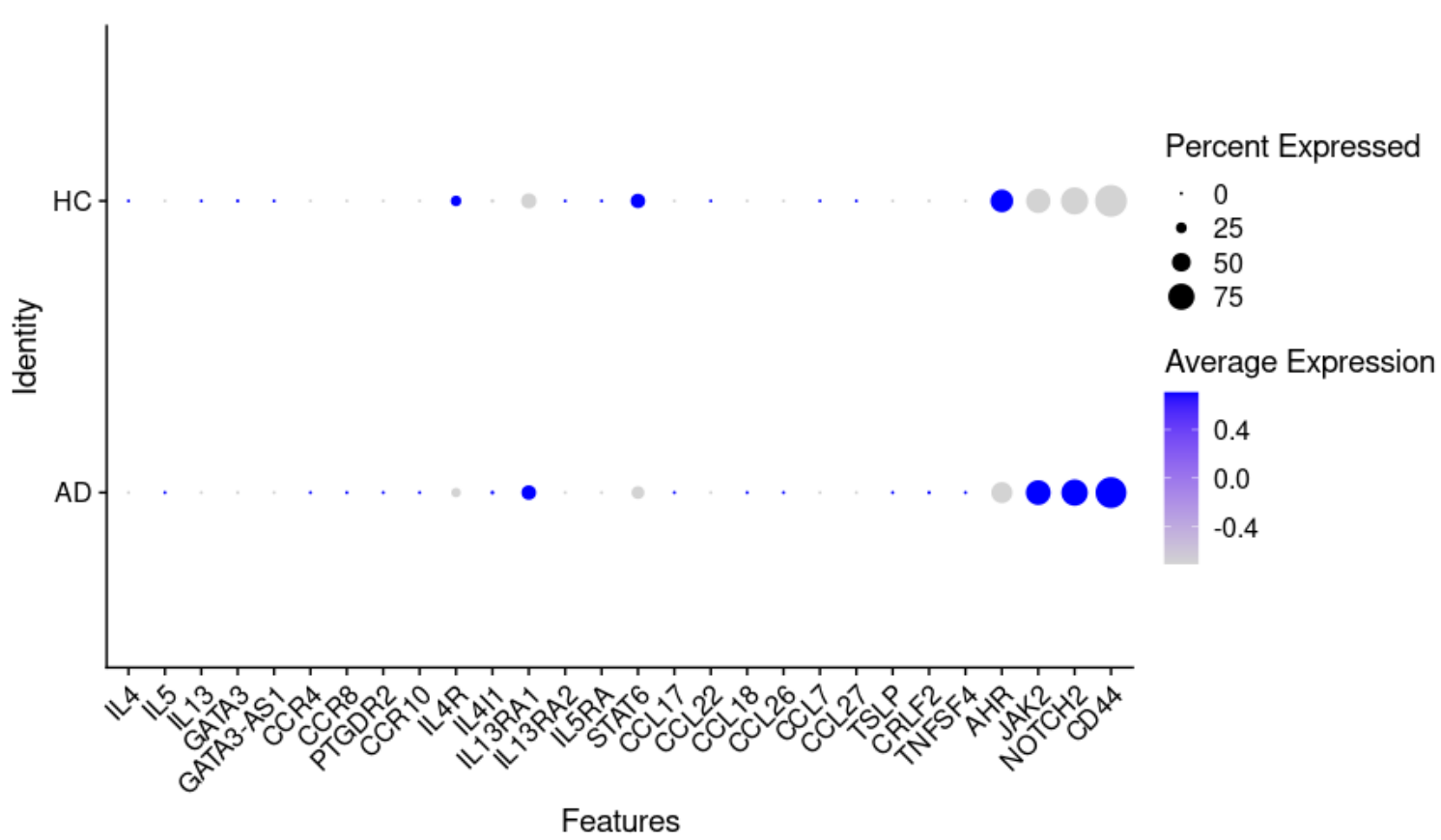

C

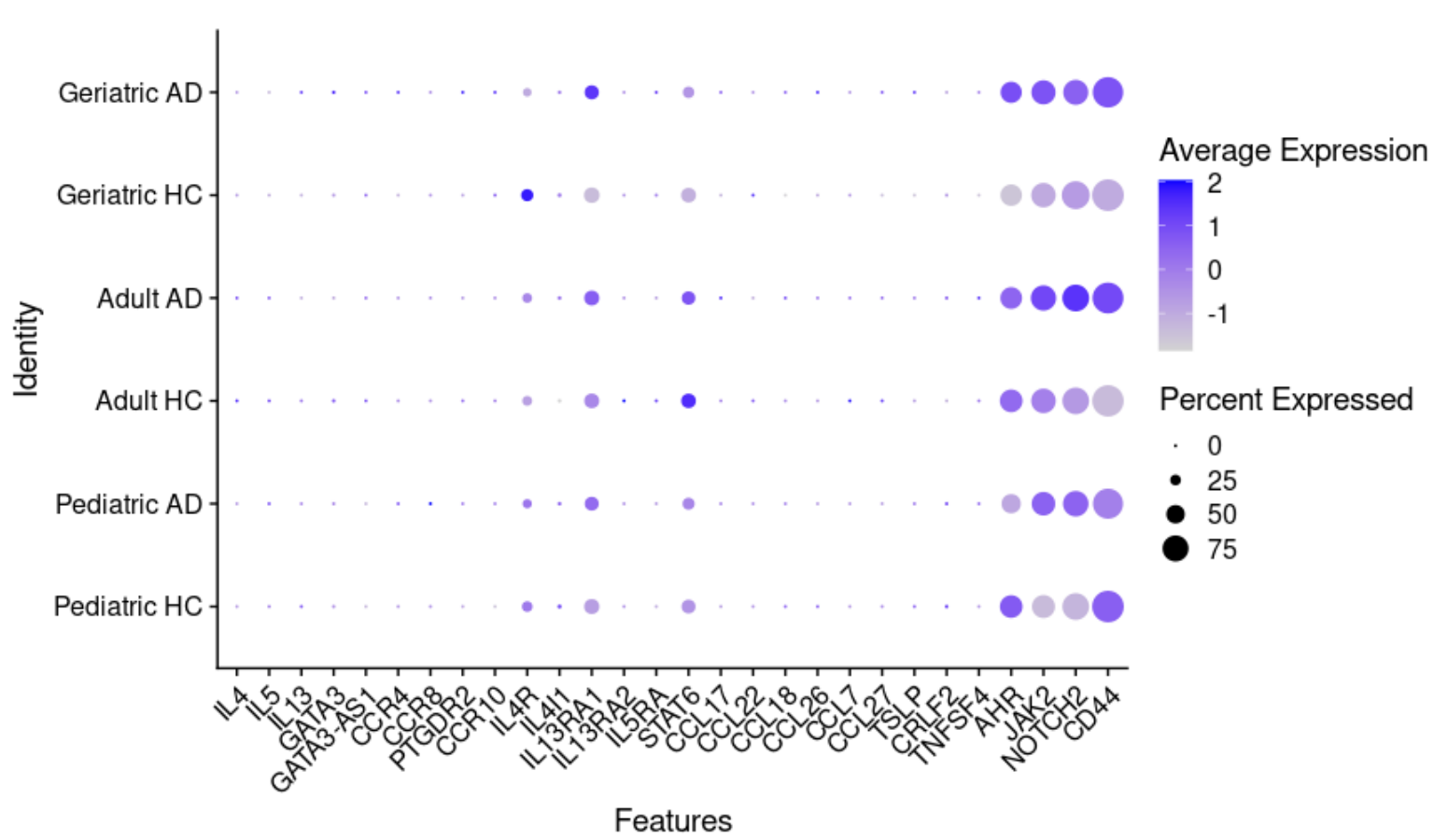

D

gene	p_val	avg_log2FC	pct.1	pct.2	p_val_adj
JAK2	9.697522e-170	0.4109343	0.701	0.685	3.267483e-165
NOTCH2	4.347433e-43	0.1916516	0.752	0.778	1.464824e-38
CD44	1.200681e-26	0.1335095	0.898	0.929	4.045575e-22
IL13RA1	9.861245e-02	0.1938659	0.371	0.389	1.000000e+00

A

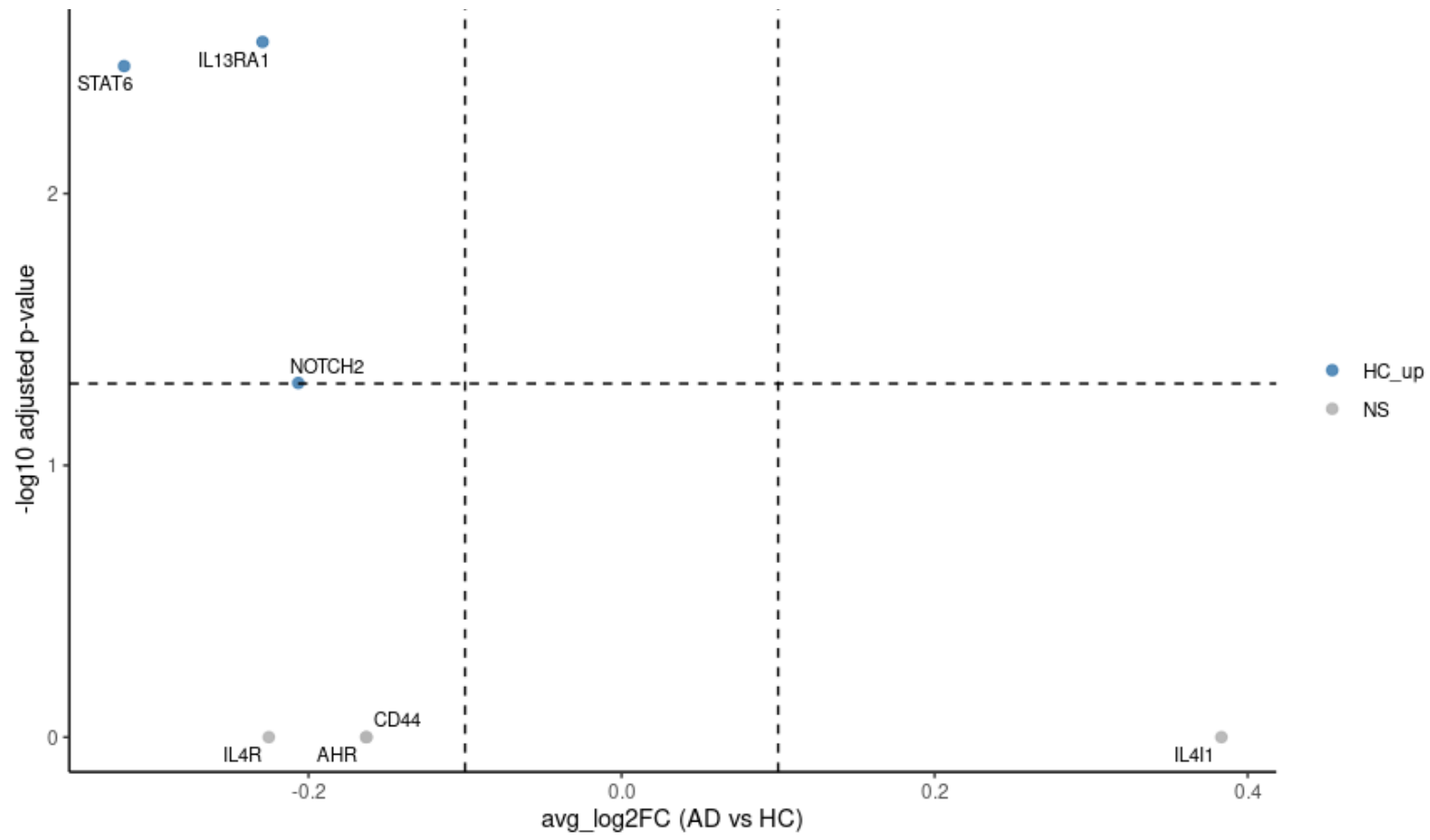

B

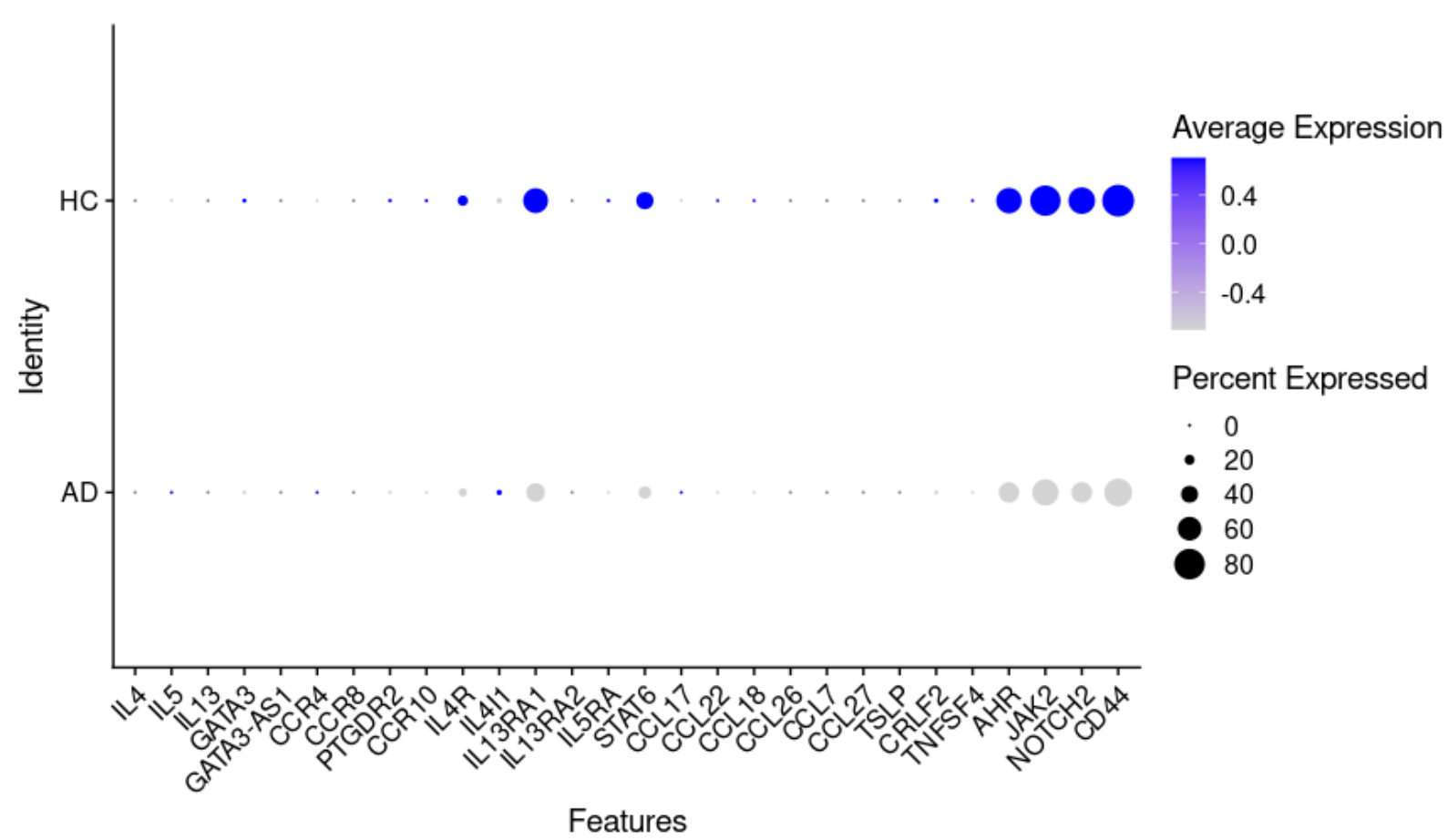

C

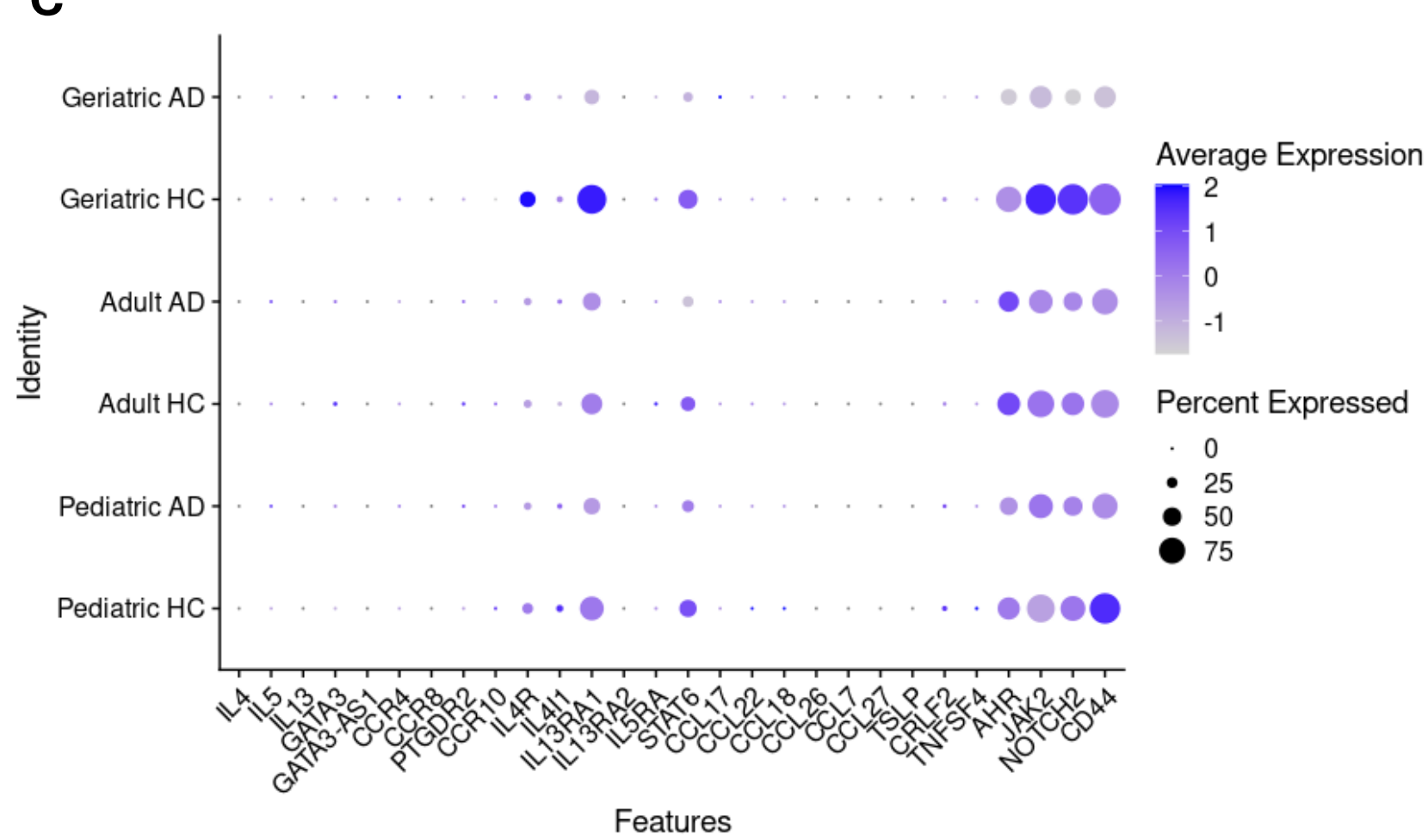

D

gene	p_val	avg_log2FC	pct.1	pct.2	p_val_adj
IL13RA1	8.209442e-08	-0.2293661	0.448	0.634	0.002766089
STAT6	1.008249e-07	-0.3177995	0.270	0.408	0.003397193
NOTCH2	1.476629e-06	-0.2065289	0.508	0.686	0.049753530
AHR	3.178221e-05	-0.1631622	0.503	0.652	1.000000000
CD44	4.333713e-04	-0.1629777	0.716	0.838	1.000000000
IL4R	5.097047e-04	-0.2253750	0.145	0.211	1.000000000
IL4I1	6.767188e-01	0.3831966	0.064	0.071	1.000000000

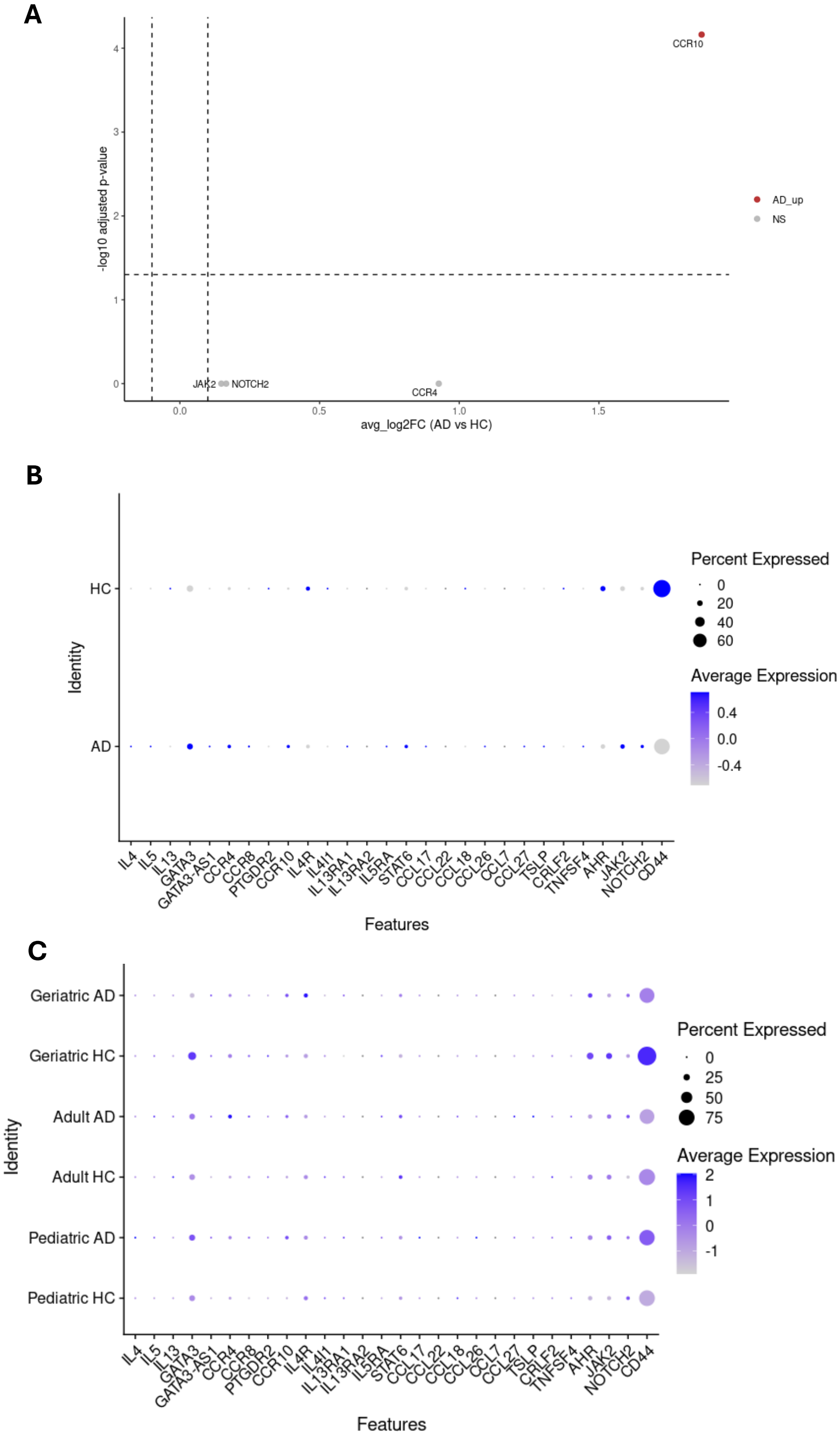

D

gene	p_val	avg_log2FC	pct.1	pct.2	p_val_adj
CCR10	2.041015e-09	1.8676306	0.076	0.035	6.876997e-05
CCR4	5.454780e-04	0.9266741	0.093	0.066	1.000000e+00
NOTCH2	1.685793e-01	0.1655536	0.081	0.093	1.000000e+00
JAK2	2.449760e-01	0.1482605	0.140	0.156	1.000000e+00

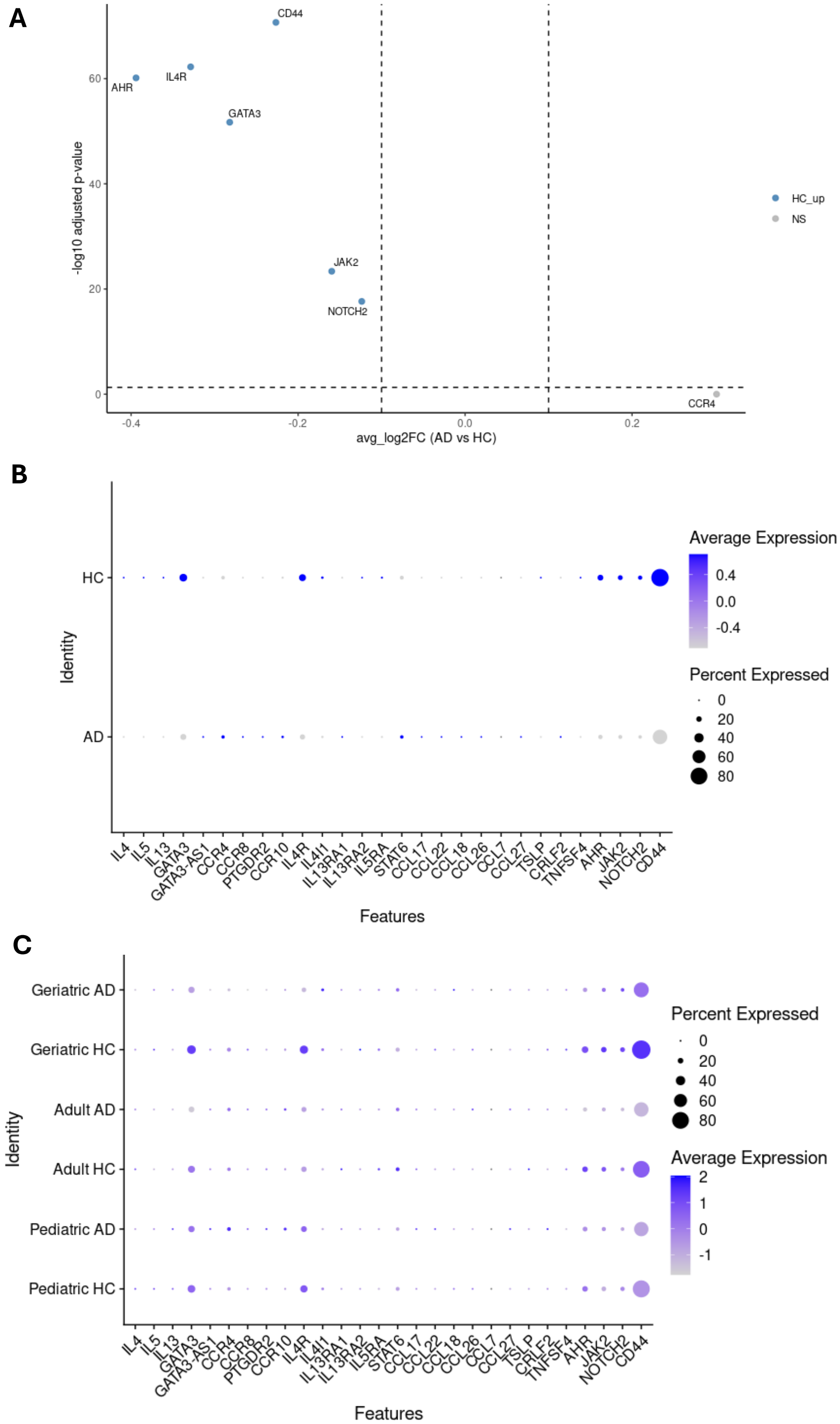

D

gene	p_val	avg_log2FC	pct.1	pct.2	p_val_adj
CD44	6.015636e-76	-0.2265680	0.678	0.833	2.026908e-71
IL4R	1.722795e-67	-0.3286827	0.184	0.277	5.804785e-63
AHR	2.142802e-65	-0.3940887	0.137	0.218	7.219958e-61
GATA3	5.876442e-57	-0.2819232	0.224	0.317	1.980008e-52
JAK2	1.269096e-28	-0.1597073	0.118	0.167	4.276092e-24
NOTCH2	6.799004e-23	-0.1237398	0.095	0.134	2.290856e-18
CCR4	8.361291e-03	0.3012480	0.087	0.098	1.000000e+00

A

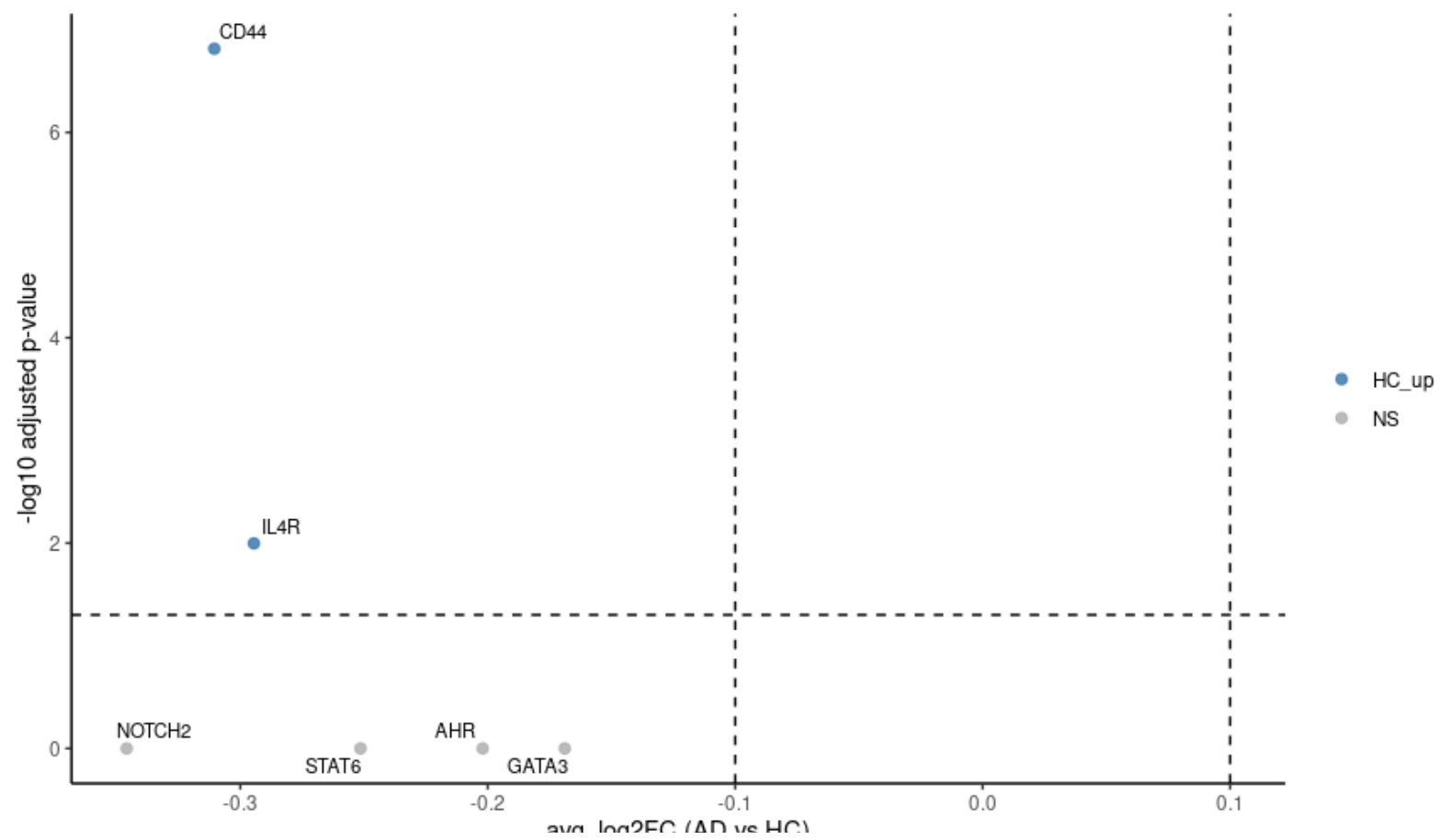

B

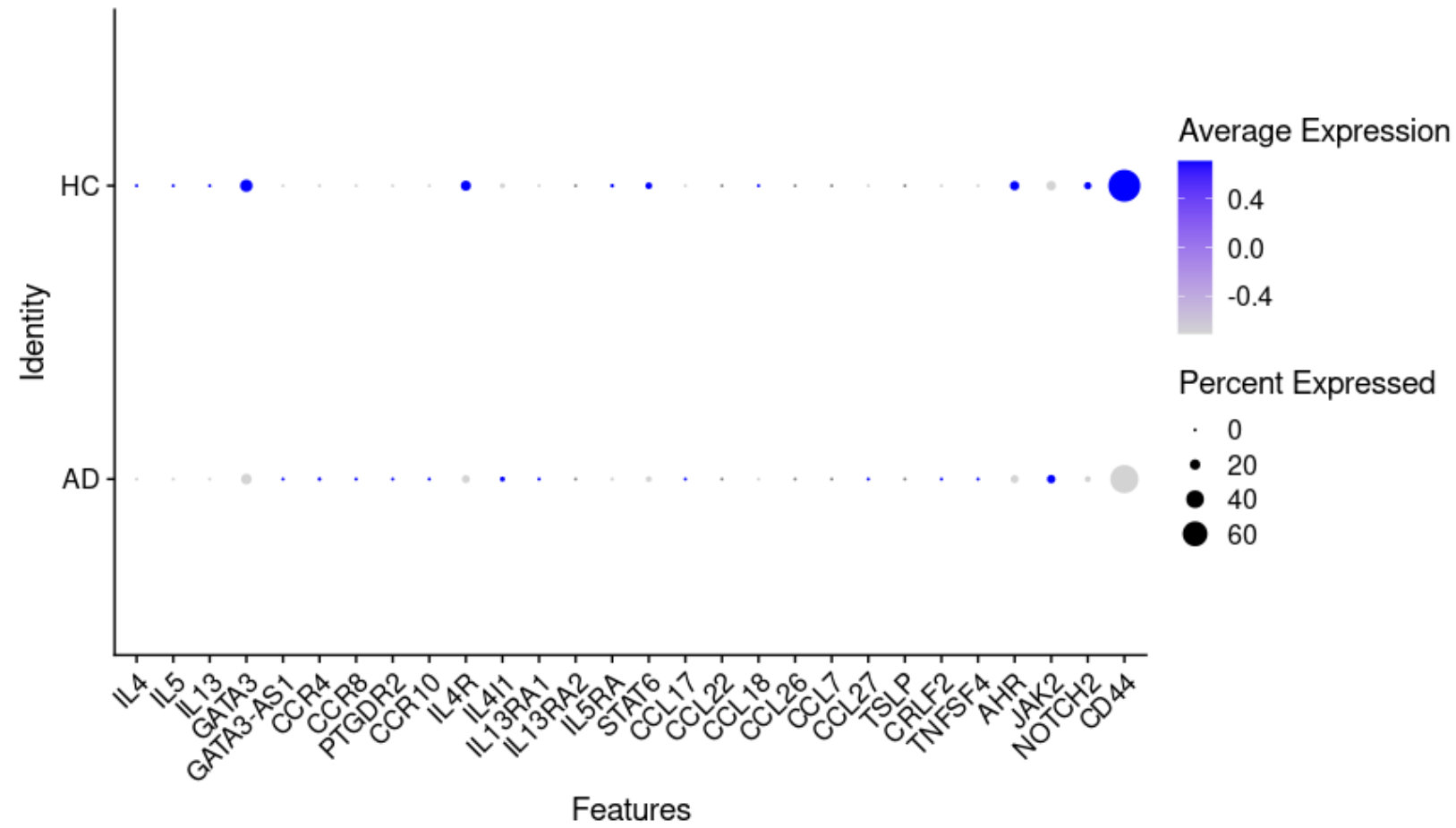

C

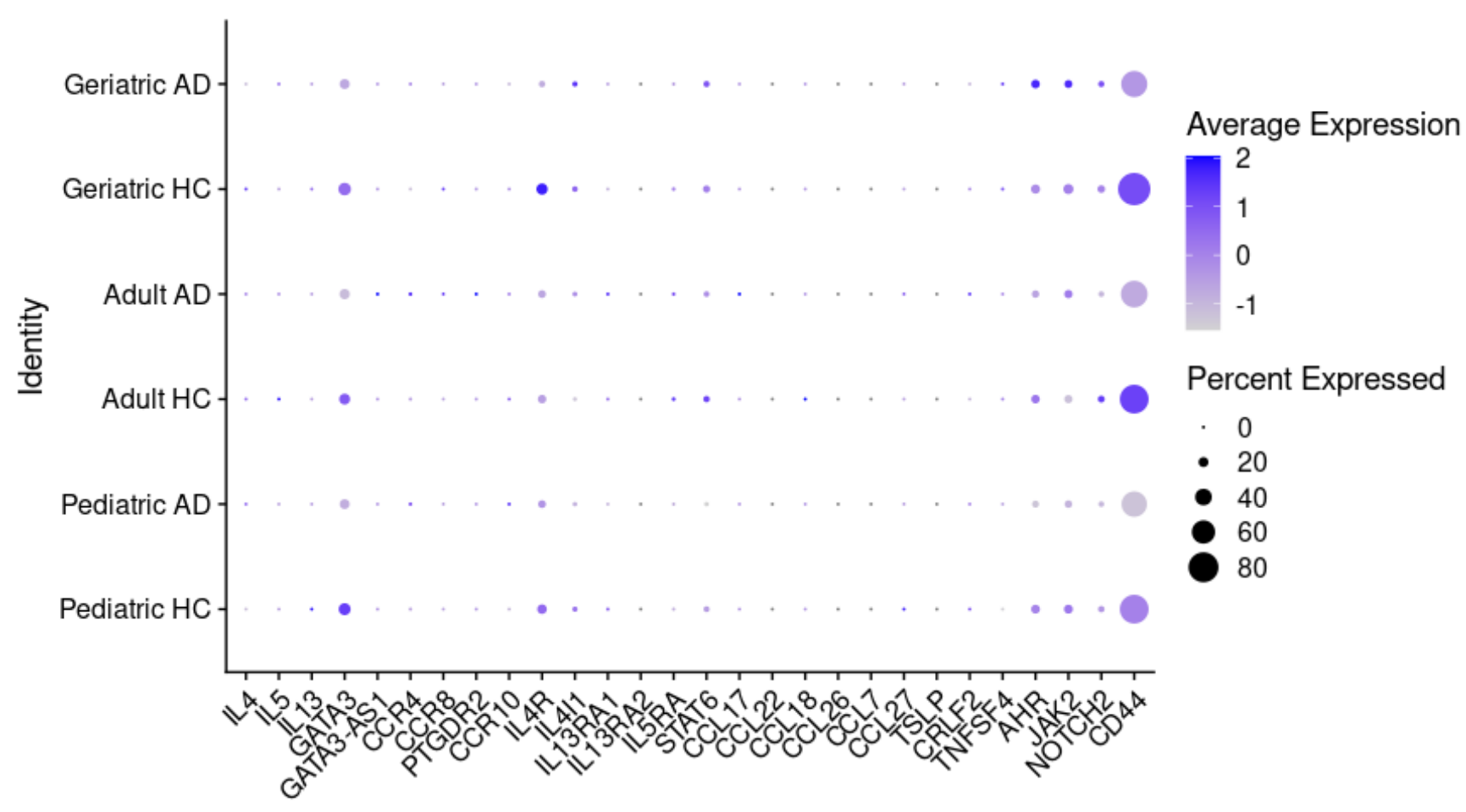

D

gene	p_val	avg_log2FC	pct.1	pct.2	p_val_adj
CD44	4.545523e-12	-0.3104779	0.690	0.798	1.531569e-07
IL4R	2.988207e-07	-0.2944922	0.130	0.194	1.006846e-02
NOTCH2	1.905887e-04	-0.3459194	0.077	0.113	1.000000e+00
AHR	1.036475e-03	-0.2020615	0.130	0.169	1.000000e+00
GATA3	1.224767e-03	-0.1688687	0.206	0.255	1.000000e+00
STAT6	3.331336e-03	-0.2514305	0.071	0.099	1.000000e+00

A

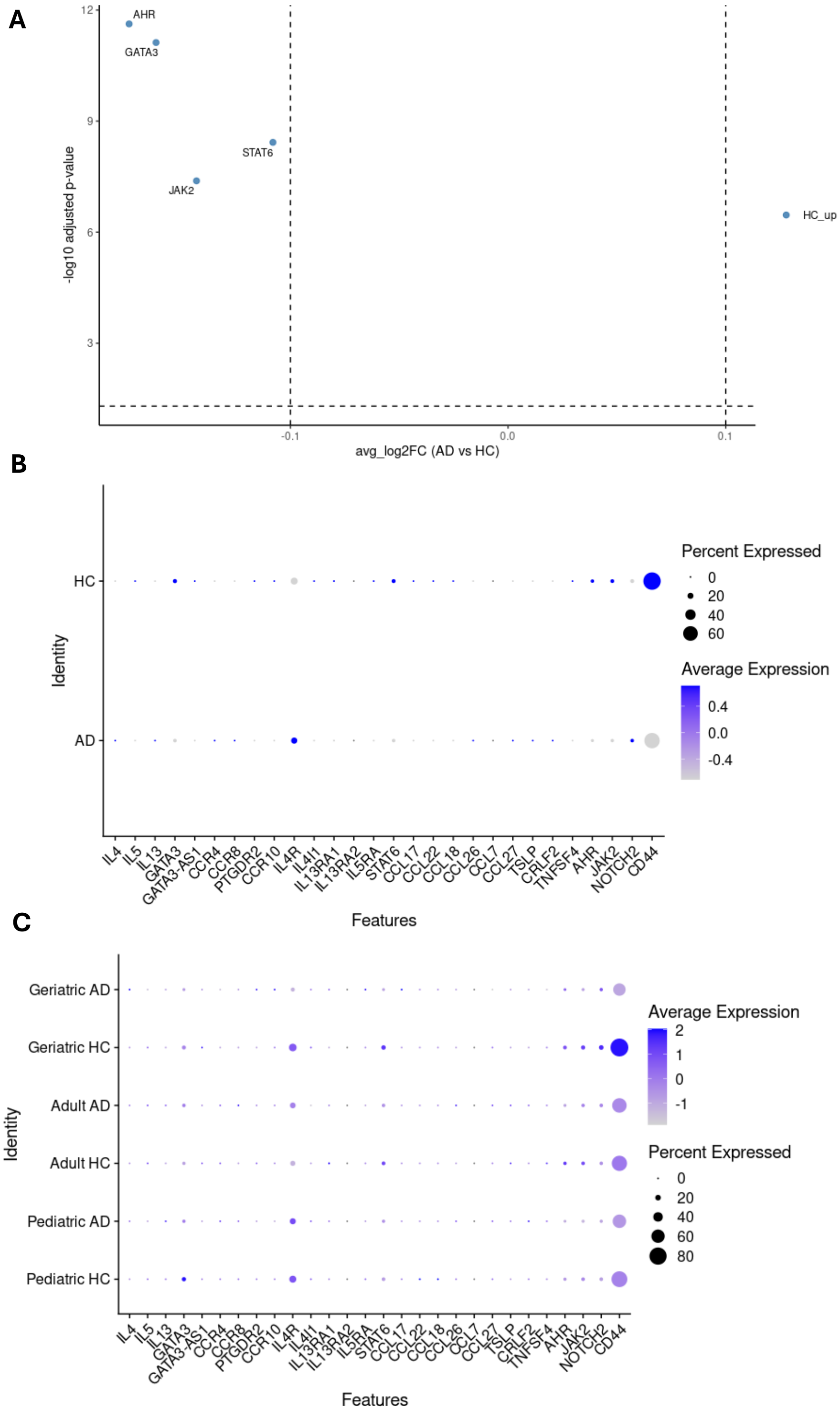

D

gene	p_val	avg_log2FC	pct.1	pct.2	p_val_adj
AHR	7.030778e-17	-0.1741590	0.060	0.083	2.368951e-12
GATA3	2.244446e-16	-0.1617750	0.085	0.111	7.562437e-12
STAT6	1.113545e-13	-0.1080659	0.082	0.105	3.751978e-09
JAK2	1.219442e-12	-0.1431861	0.077	0.099	4.108789e-08

A

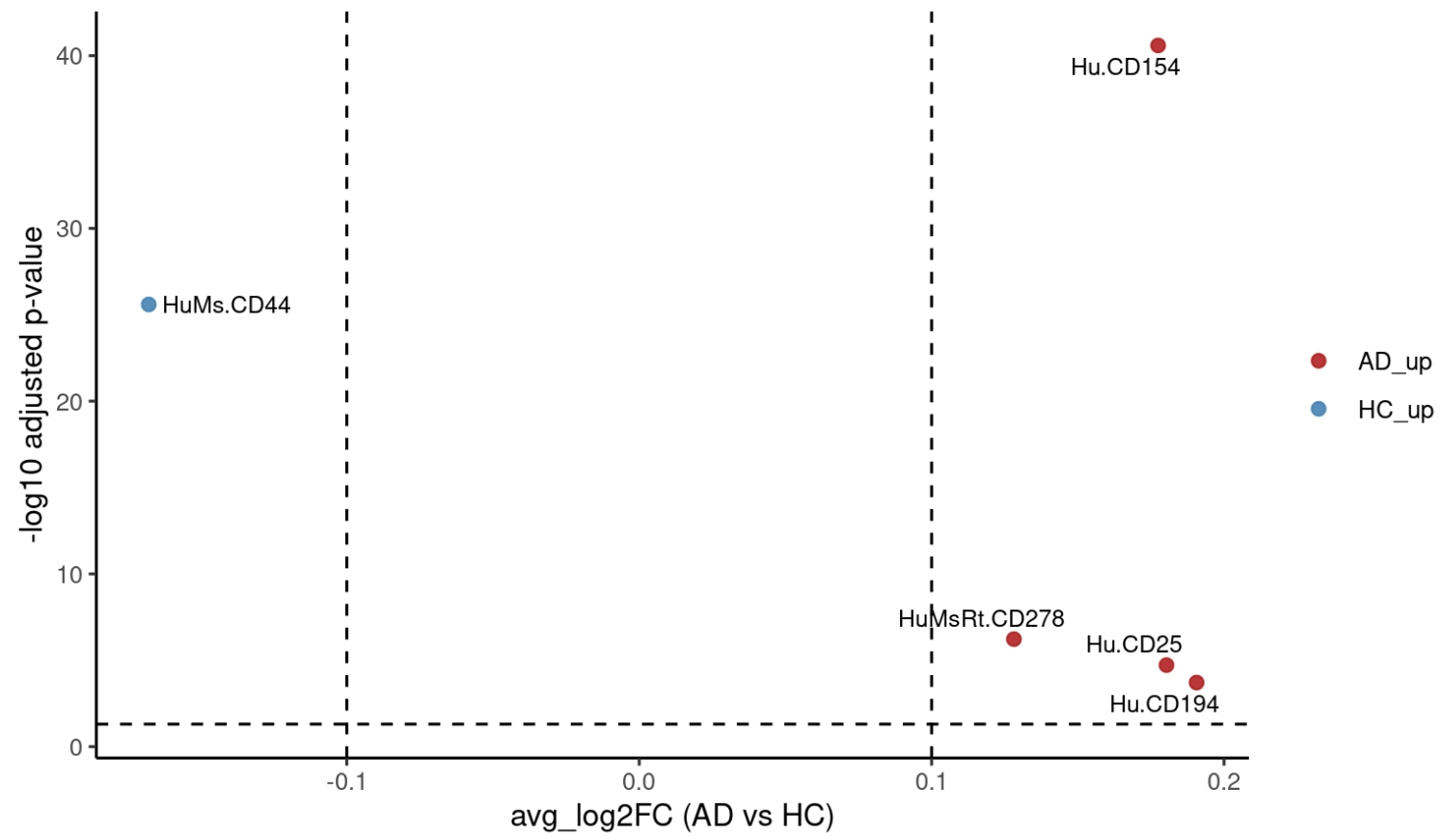

B

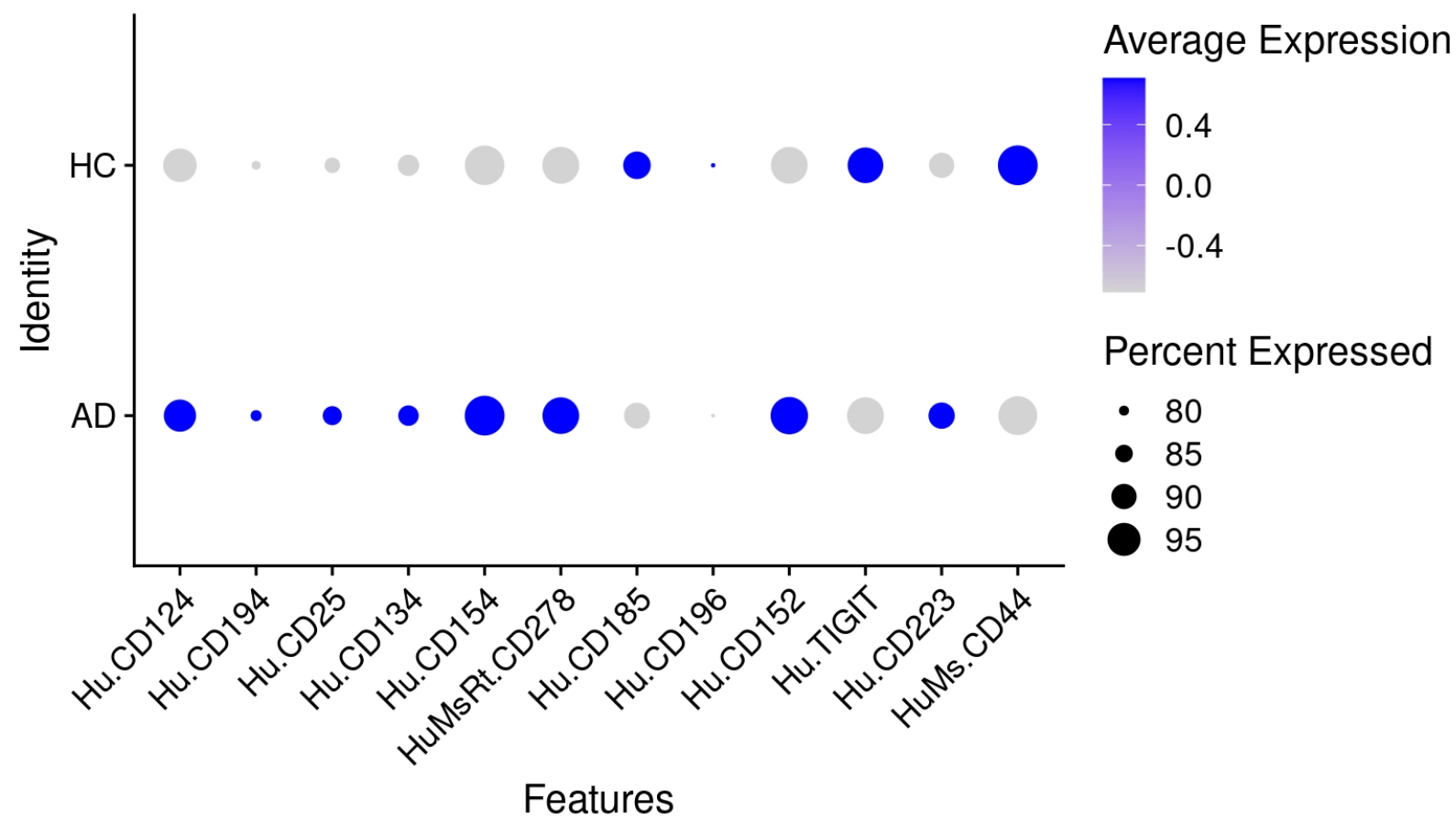

C

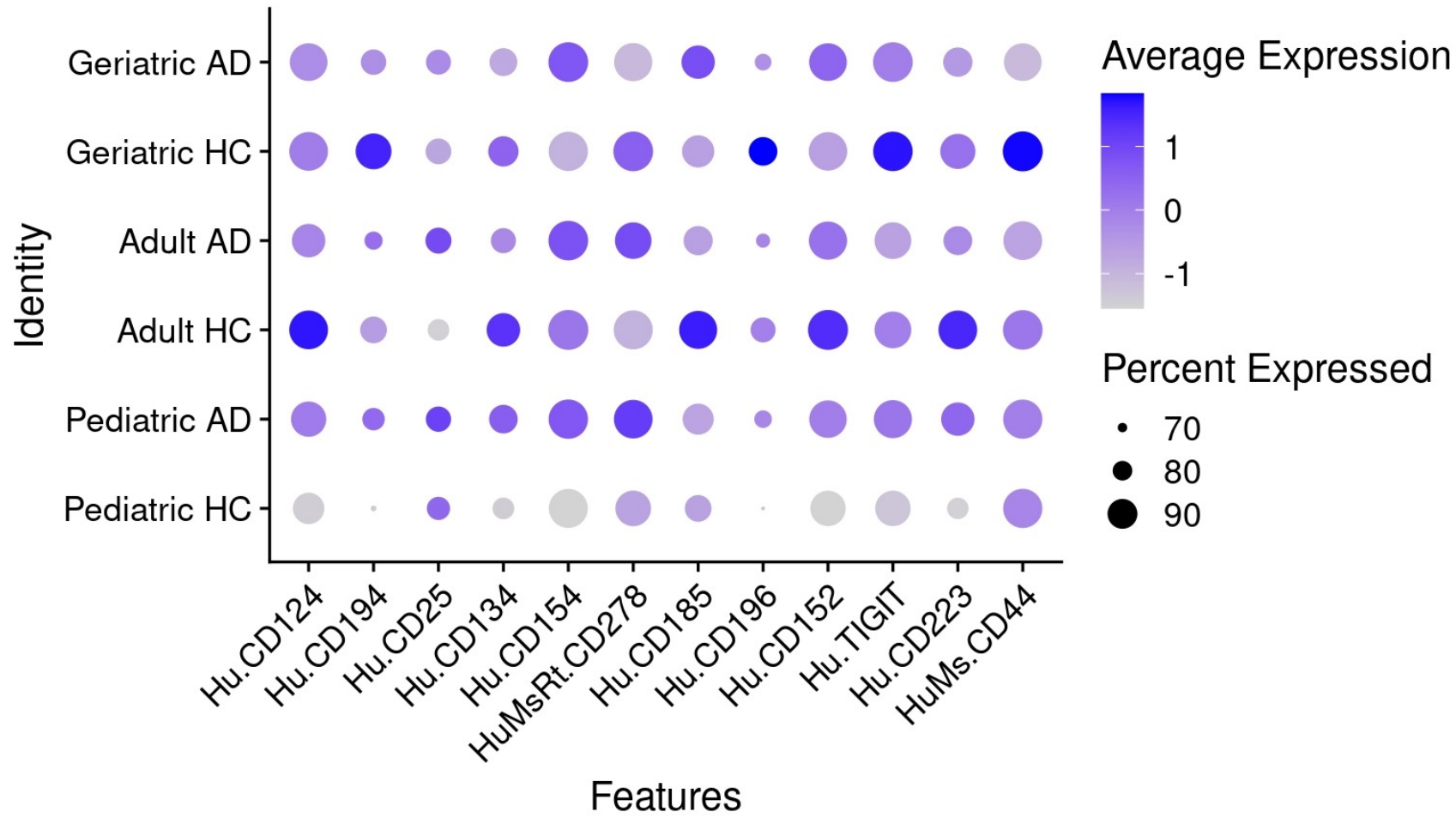

D

protein	p_val	avg_log2FC	pct.1	pct.2	p_val_adj
Hu.CD154	1.831183e-43	0.1774547	0.994	0.992	2.563656e-41
HuMs.CD44	1.806729e-28	-0.1677206	0.988	0.994	2.529421e-26
HuMsRt.CD278	4.281478e-09	0.1281485	0.973	0.975	5.994070e-07
Hu.CD25	1.367138e-07	0.1803193	0.859	0.836	1.913993e-05
Hu.CD194	1.397738e-06	0.1906044	0.806	0.793	1.956834e-04
