# Supplementary material for: Immunological differences in atopic dermatitis across age groups: insights from single-cell multi-omics
Source: J Transl Med. 2026 Jun 1;24:1004. doi: 10.1186/s12967-026-08288-7 (PMC13445865; doi:10.1186/s12967-026-08288-7)
Supplement: Supplementary file 4 [file 12967_2026_8288_MOESM7_ESM.pdf]

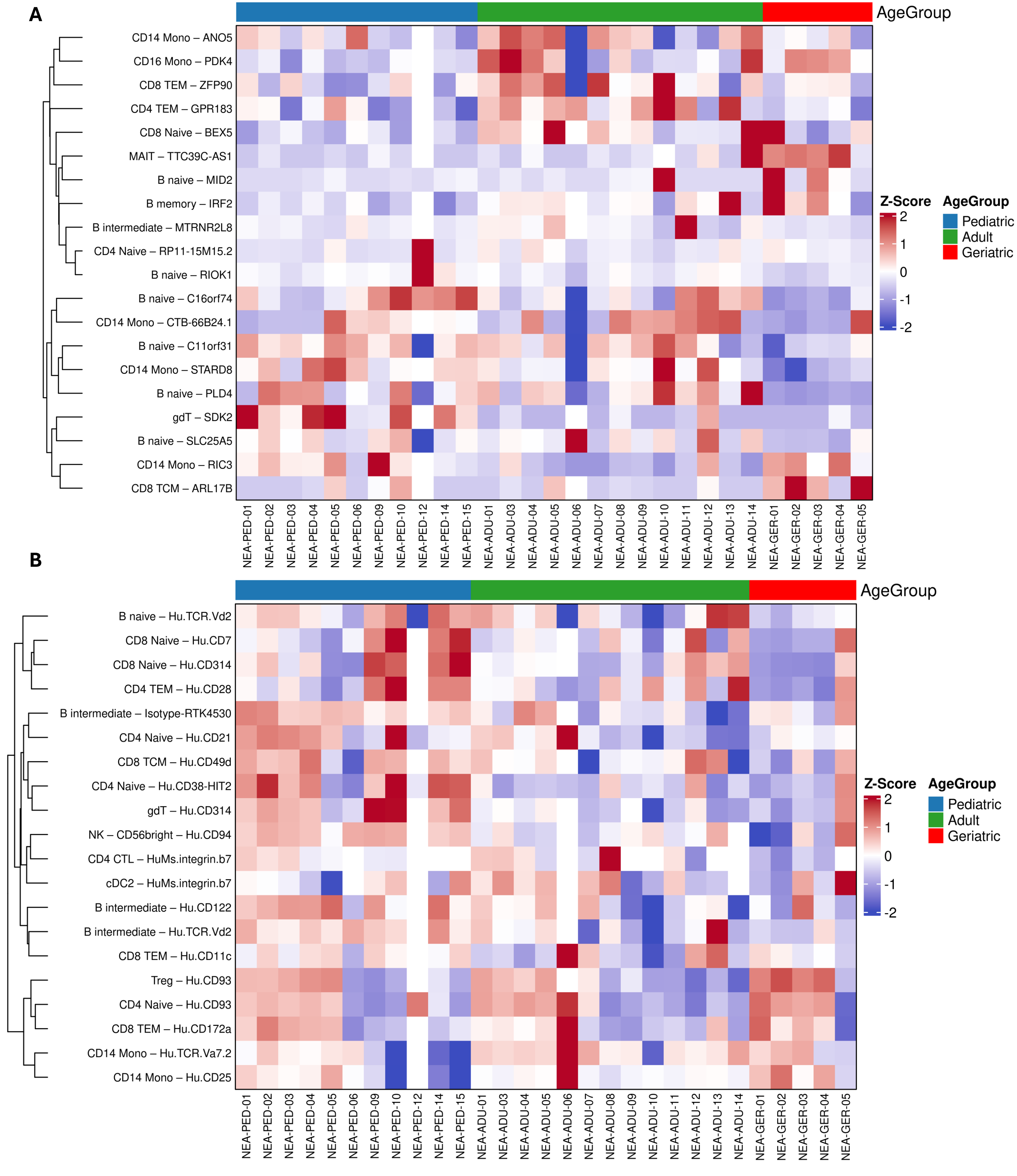

A
AgeGroup
CD14 Mono – ANO5
CD16 Mono – PDK4
CD8 TEM – ZFP90
CD4 TEM – GPR183
CD8 Naive – BEX5
MAIT – TTC39C-AS1
B naive – MID2
B memory – IRF2
B intermediate – MTRNR2L8
CD4 Naive – RP11-15M15.2
B naive – RIOK1
B naive – C16orf74
CD14 Mono – CTB-66B24.1
B naive – C11orf31
CD14 Mono – STARD8
B naive – PLD4
gdT – SDK2
B naive – SLC25A5
CD14 Mono – RIC3
CD8 TCM – ARL17B
NEA-PED-01
NEA-PED-02
NEA-PED-03
NEA-PED-04
NEA-PED-05
NEA-PED-06
NEA-PED-09
NEA-PED-10
NEA-PED-12
NEA-PED-14
NEA-PED-15
NEA-ADU-01
NEA-ADU-03
NEA-ADU-04
NEA-ADU-05
NEA-ADU-06
NEA-ADU-07
NEA-ADU-08
NEA-ADU-09
NEA-ADU-10
NEA-ADU-11
NEA-ADU-12
NEA-ADU-13
NEA-ADU-14
NEA-GER-01
NEA-GER-02
NEA-GER-03
NEA-GER-04
NEA-GER-05
Z-Score
2
1
0
-1
-2
AgeGroup
Pediatric
Adult
Geriatric
B
AgeGroup
B naive – Hu.TCR.Vd2
CD8 Naive – Hu.CD7
CD8 Naive – Hu.CD314
CD4 TEM – Hu.CD28
B intermediate – Isotype-RTK4530
CD4 Naive – Hu.CD21
CD8 TCM – Hu.CD49d
CD4 Naive – Hu.CD38-HIT2
gdT – Hu.CD314
NK – CD56bright – Hu.CD94
CD4 CTL – HuMs.integrin.b7
cDC2 – HuMs.integrin.b7
B intermediate – Hu.CD122
B intermediate – Hu.TCR.Vd2
CD8 TEM – Hu.CD11c
Treg – Hu.CD93
CD4 Naive – Hu.CD93
CD8 TEM – Hu.CD172a
CD14 Mono – Hu.TCR.Va7.2
CD14 Mono – Hu.CD25
NEA-PED-01
NEA-PED-02
NEA-PED-03
NEA-PED-04
NEA-PED-05
NEA-PED-06
NEA-PED-09
NEA-PED-10
NEA-PED-12
NEA-PED-14
NEA-PED-15
NEA-ADU-01
NEA-ADU-03
NEA-ADU-04
NEA-ADU-05
NEA-ADU-06
NEA-ADU-07
NEA-ADU-08
NEA-ADU-09
NEA-ADU-10
NEA-ADU-11
NEA-ADU-12
NEA-ADU-13
NEA-ADU-14
NEA-GER-01
NEA-GER-02
NEA-GER-03
NEA-GER-04
NEA-GER-05
Z-Score
2
1
0
-1
-2
AgeGroup
Pediatric
Adult
Geriatric
