# Supplementary material for: Immunological differences in atopic dermatitis across age groups: insights from single-cell multi-omics
Source: J Transl Med. 2026 Jun 1;24:1004. doi: 10.1186/s12967-026-08288-7 (PMC13445865; doi:10.1186/s12967-026-08288-7)
Supplement: Supplementary file 5 [file 12967_2026_8288_MOESM10_ESM.docx]

## Additional Files

Additional File 1

- File format: .xls
- Title of data: Supplementary Table 1. Subject clinical and demographic characteristics.

Additional File 2

- File format: .xls
- Title of data: Supplementary Table 2. 140-plex TotalSeq-B antibody panel.
- Description of data: List of 140 antibodies included in the TotalSeq-B panel used for cell staining.

Additional File 3

- File format: .pdf
- Title of data: Supplementary Figure 1. Identification of donor-restricted outlier cluster prior to integration.
- Description of data:
  - (A) Unintegrated UMAP colored by Azimuth-based cell annotations (*predicted.celltype.l2*), showing major immune populations.
  - (B) Unintegrated UMAP colored by *seurat_clusters*, highlighting a discrete island cluster adjacent to the CD14+ monocyte population.
  - (C) UMAP highlighting the three excluded samples, demonstrating their co-localization within the island cluster.
  - (D) Bar plot showing sample-level contributions to the island cluster, indicating disproportionate contribution from a small subset of samples.

Additional File 4

- File format: .pdf
- Title of data: Supplementary Figure 2. Quality control and compositional analysis of excluded samples.
- Description of data:
  - (A) QC distributions (nFeature_RNA, nCount_RNA, and mitochondrial percentage) for the three excluded samples.
  - (B) QC distributions for cells from the excluded samples outside the identified island cluster.
  - (C) QC distributions for cells from the excluded samples within the island cluster.
  - (D) Comparison of QC metrics between excluded samples and all other samples, showing no gross differences in nFeature_RNA, nCount_RNA, or mitochondrial percentage.
  - (E) Cell type composition of the excluded samples, showing relative proportions of CD14+ monocytes and other cell types.

Additional File 5

- File format: .pdf
- Title of data: Supplementary Figures 3–9. Differential expression analyses across key immune cell types.
- Description of data:

Differential gene expression (DEG) and differential protein expression (DEP) analyses for selected immune cell types. Each figure corresponds to one cell type and includes:

(A) Volcano plot of DEG/Ps comparing all AD versus all HC (min.pct = 0.05, log2FC threshold = 0.10).

(B) Dot plot showing expression of DEG/Ps in AD versus HC.

(C) Dot plot showing expression of DEG/Ps across all six subgroups (pediatric AD/HC, adult AD/HC, geriatric AD/HC).

(D) Table of significant DEG/Ps with corresponding statistics (p-value, adjusted p-value, log2 fold change, and percent expression).

Figure mapping: Supplementary Figure 3, CD14+ monocytes (DEG); Supplementary Figure 4, cDC2 (DEG); Supplementary Figure 5, Tregs (DEG); Supplementary Figure 6, CD4+ TCM (DEG); Supplementary Figure 7, CD4+ TEM (DEG); Supplementary Figure 8, CD4+ naïve (DEG); Supplementary Figure 9, Tregs (DEP).

Additional File 6

- File format: .xls
- Title of data: Supplementary Table 3. Pathway enrichment analysis results from g:Profiler.
- Description of data: Results of pathway enrichment analysis performed using g:Profiler on differentially expressed genes. The table includes enriched terms by cell type, along with associated statistical metrics, including p-values, adjusted p-values, term size, query size, intersection size, precision, recall, and source database annotations. Pathways shown were filtered to highlight Th2-related and immune-relevant biological processes.

Additional File 7

- File format: .pdf
- Title of data: Supplementary Figure 10. Z-scored average expression of top stable gene and protein features.
- Description of data: Heatmaps showing Z-scored average expression for (A) the top 20 stable genes and (B) the top 20 stable proteins.

Additional File 8

- File format: .xls
- Title of data: Supplementary Table 4. Transcriptomic functional groups.
- Description of data: Mapping of top stable genes to their functional groups used in Figure 6

Additional File 9

- File format: .xls
- Title of data: Supplementary Table 5. Proteomic functional groups.
- Description of data: Mapping of top stable proteins to their functional groups used in Figure 6.
